# Effect of Natural Adenylcyclase/cAMP/CREB Signalling Activator Forskolin against Intra-Striatal 6-OHDA-Lesioned Parkinson’s Rats: Preventing Mitochondrial, Motor and Histopathological Defects

**DOI:** 10.3390/molecules27227951

**Published:** 2022-11-17

**Authors:** Metab Alharbi, Abdulrahman Alshammari, Gurpreet Kaur, Sanjeev Kalra, Sidharth Mehan, Manisha Suri, Swesha Chhabra, Nitish Kumar, Wael A. Alanazi, Aliah R. Alshanwani, Abdullah Hamed AL-Ghamdi, Acharan S. Narula, Reni Kalfin

**Affiliations:** 1 Department of Pharmacology and Toxicology, College of Pharmacy, King Saud University, Post Box 2455, Riyadh 11451, Saudi Arabia; 2 Department of Pharmacology, Rajendra Institute of Technology and Sciences, Hisar Road, 4th Mile Stone, Sirsa, Haryana 125055, India; 3 Division of Neuroscience, Department of Pharmacology, ISF College of Pharmacy (An Autonomous College), Moga 142001, Punjab, India; 4 Physiology Department, College of Medicine & King Khalid University Hospital, King Saud University, Riyadh 12372, Saudi Arabia; 5 Pharmaceutical Care Department, Namerah General Hospital, Ministry of Health, Namerah 65439, Saudi Arabia; 6 Narula Research, LLC, 107 Boulder Bluff, Chapel Hill, NC 27516, USA; 7 Institute of Neurobiology, Bulgarian Academy of Sciences, Acad. G. Bonchev St., Block 23, 1113 Sofia, Bulgaria; 8 Department of Healthcare, South-West University “NeofitRilski”, Ivan Mihailov St. 66, 2700 Blagoevgrad, Bulgaria

**Keywords:** adenylcyclase, CREB, forskolin, mitochondrial abnormalities, Parkinson’s disease

## Abstract

Parkinson’s disease (PD) is characterised by dopaminergic neuronal loss in the brain area. PD is a complex disease that deteriorates patients’ motor and non-motor functions. In experimental animals, the neurotoxin 6-OHDA induces neuropathological, behavioural, neurochemical and mitochondrial abnormalities and the formation of free radicals, which is related to Parkinson-like symptoms after inter-striatal 6-OHDA injection. Pathological manifestations of PD disrupt the cAMP/ATP-mediated activity of the transcription factor CREB, resulting in Parkinson’s-like symptoms. Forskolin (FSK) is a direct AC/cAMP/CREB activator isolated from *Coleus forskohlii* with various neuroprotective properties. FSK has already been proven in our laboratory to directly activate the enzyme adenylcyclase (AC) and reverse the neurodegeneration associated with the progression of Autism, Multiple Sclerosis, ALS, and Huntington’s disease. Several behavioural paradigms were used to confirm the post-lesion effects, including the rotarod, open field, grip strength, narrow beam walk (NBW) and Morris water maze (MWM) tasks. Our results were supported by examining brain cellular, molecular, mitochondrial and histopathological alterations. The FSK treatment (15, 30 and 45 mg/kg, orally) was found to be effective in restoring behavioural and neurochemical defects in a 6-OHDA-induced experimental rat model of PD. As a result, the current study successfully contributes to the investigation of FSK’s neuroprotective role in PD prevention via the activation of the AC/cAMP/PKA-driven CREB pathway and the restoration of mitochondrial ETC-complex enzymes.

## 1. Introduction

Parkinson’s disease (PD) is a neurological condition that causes neurodegeneration as well as motor impairments (tremors, muscle weakness, akinesia and postural instability) [1]. Due to dopamine (DA) deficiency, Parkinson’s disease (PD) is characterised by Lewy bodies (eosinophilic protein depositions) in the substantia nigra pars compacta (SNPc) [2,3]. PD affects 1–2% of persons over the age of 60, rising to 4% by the age of 80. Parkinson’s disease (PD) is distinguished by the progressive loss of dopaminergic substantia nigra neurons that project directly to the striatum [3]. According to recent findings, 0.58 million patients in India have Parkinson’s disease [4]. Basal (BG) dysfunction causes memory and olfactory impairment, sleep trouble and neuropsychiatric symptoms such as depression, hallucinations and dementia [5]. These characteristics may result from extrapyramidal Parkinson’s disease (PD), as well as chronic inflammation and oxidative stress [6,7]. Several other genes, including SNCA, Parkin (PARK2), NR4A2 (NURR1), PARK3 and PARK4 play important roles in the progression of PD [8].

PD has various neuropathological features, including brain stem degeneration, mitochondrial ETC-complex deficits, cellular protein transport abnormalities and excitotoxicity. Mitochondrial failure causes decreased ATP synthesis, increased DNA mutation and an intracellular calcium imbalance [9].

Dopaminergic neurodegeneration is linked to neurological complications in experimental PD animals. Toxins such as 6-hydroxydopamine (6-OHDA), methyl-4-phenyl-1,2,3,6-tetrahydropyridine (MPTP) and rotenone are used in animal research to induce neuropathological dysfunctions and PD-like symptoms [10]. Dopaminergic neurodegeneration was induced using 6-OHDA, one of these compounds [11]. Rats given 8 μg/2 μL (AP—0.5 mm; ML—2.5 mm; DV—5 mm) and mice given 0.5 μL/min (AP—0.9 mm; ML—1.8 mm; DV—3.0 mm) demonstrated similar behavioural and biochemical abnormalities as seen in PD patients [12,13]. ICV-induced 6-OHDA causes an increase in oxidative stress, which damages dopaminergic neurons and interferes with the dopaminergic nigrostriatal pathway [14]. It has been observed that unilateral intra-striatal injection of 6-OHDA causes neurochemical (glutamatergic, cholinergic, tryptaminergic, GABAergic, noradrenergic and adrenergic) and behavioural (aphagia, adipsia, paradoxical kinesia, tremulous jaw movement, epileptic seizures) abnormalities comparable to those seen in PD [15].

Brain-derived neurotrophic factor (BDNF) is a cAMP-regulated protein necessary for neuron survival, synaptic plasticity, excitotoxicity reduction and amyloid-β toxicity [13,16,17]. Increasing cAMP levels is beneficial for cholinergic, adrenergic and dopaminergic neurons in the striatum, hypothalamus and substantia nigra (SN), as well as cellular energy and neurotransmitter synthesis and release [18]. cAMP-dependent CREB phosphorylation increases LTP, anti-apoptotic gene regulation, mitochondrial biogenesis and BDNF expression.

Several natural substances have been studied for their potential to prevent Parkinson’s disease by altering neuronal cellular and molecular processes [19]. Several treatments increase the cAMP/PKA/CREB pathways to protect against neurodegenerative illnesses (stroke, depression, schizophrenia, Alzheimer’s and HD) [20,21]. In the current study, we used Forskolin (FSK), derived from *Coleus forskohlii*, to treat CNS diseases such as insomnia, depression and seizures. In addition to its antioxidant and anti-inflammatory properties, it also has spasmolytic, sedative, anti-convulsive and asthmatic effects [22]. FSK reduces mitochondrial dysfunction in cardiomyopathy, asthma, glaucoma, hypertension, hair loss, cancer and obesity [23].

Our latest laboratory research on FSK discovered that it is vital in preventing oligodendrocyte cell degeneration and death in neurodegenerative illnesses such as multiple sclerosis and amyotrophic lateral sclerosis (ALS) by activating the cAMP and CREB pathways [24,25]. As a result, by directly activating the enzyme adenylcyclase (AC), FSK is essential to reverse OHDA-induced neuronal death [13]. FSK activates adenylcyclase, and its neuroprotective effect in mitochondrial dysfunction is mediated through CREB [26,27]. The natural AC activator Coleus Forskohlii (FSK) has been shown to protect against neurodegenerative diseases by modulating the cAMP/CREB [28], BDNF [29], PI3K/Akt and ERK1/2 pathways [30].

The AC/cAMP/CREB pathway lowers inflammatory cytokines [31], plasminogen activating factor (PAF) [32], mast cell degranulation [33] and oxidative stress [34]. In the context of these observations, we conducted research on the neuroprotective effects of FSK on rats with 6-OHDA-induced Parkinson’s disease (PD)-like symptoms via activating the AC/cAMP/CREB pathway.

## 2. Results

### 2.1. Behavioural Parameters

#### 2.1.1. FSK Improved Memory and Cognition in an Experimental Model of PD

**Figure 1:**
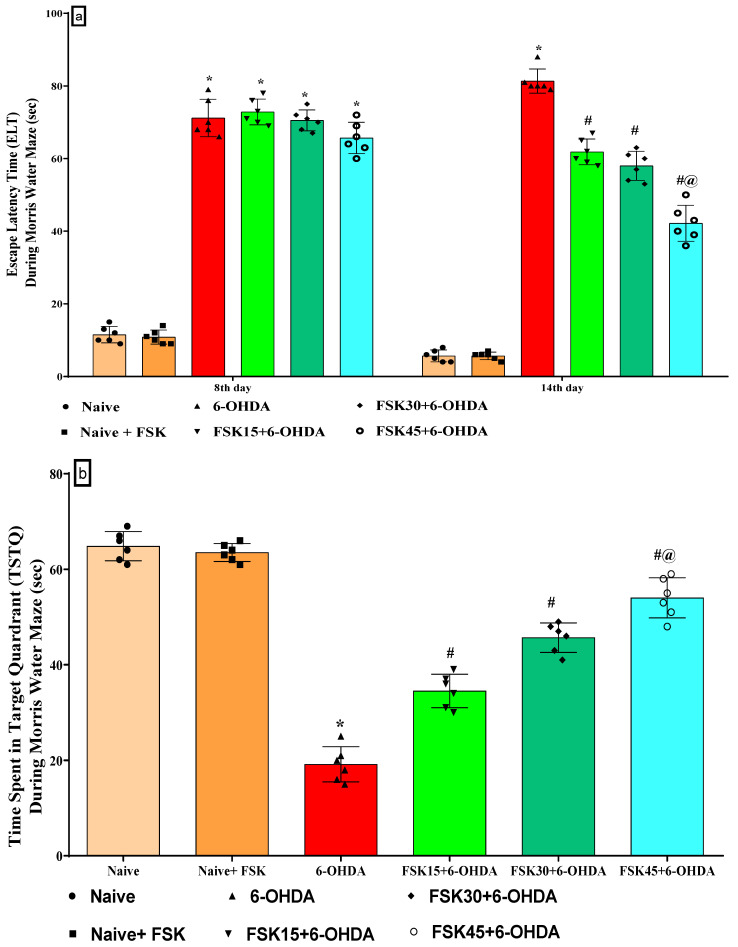
(**a**) FSK improved escape latency time using the Morris water maze in the experimental model of PD. *Data provided as mean ± SD (n =* 6*) followed by ANOVA (two-way) with post-hoc Bonferroni test*. * 6-OHDA (*p* < 0.01) compared with naive *and naive + FSK*; *# FSK15, FSK30, FSK45 (p < 0.01)* compared with 6-*OHDA*; *#@ FSK45 (p <* 0.01*)* compared with *FSK30, FSK15*. (**b**) FSK improved TSTQ using the Morris water maze in the experimental model of PD. *Data provided as mean ± SD (n = *6*) followed by ANOVA (one-way) with post-hoc Tukey’s test.* * 6-OHDA (*p* < 0.01) compared with naive *and naive + FSK*; *#FSK15, FSK30, FSK45 (p < *0.01*)* compared with *6-OHDA*; *#@ FSK45 (p <* 0.01*)* compared with *FSK30, FSK15*.

The learning and memory was evaluated on days 8, 14 and 22. There was no significant difference between the naive and naive + FSK (45 mg/kg p.o.) groups. In contrast to the naive and naive + FSK groups, there was a progressive rise in ELT after the chronic injection of 6-OHDA (8 μg/2 μL/unilateral). In addition, the chronic administration of FSK at doses of 15, 30 and 45 mg/kg decreased the ELT dose. According to the protocol, escape latency tests (ELT) in the MWM were conducted on days 8 and 14, and there was a dose-dependent difference when compared with 6-OHDA-treated group [F_5,30_ = 37.64; ANOVA (two-way); *p* < 0.01] (Figure 1a). A high dose of FSK (45 mg/kg) lowers the ELT and is more effective in restoring 6-OHDA, which results in rats developing PD. TSTQ was performed on the 21st day of treatment, but no statistically significant changes were seen between the groups, i.e., naive and naive + FSK. The chronic treatment of 6-OHDA (8 μg/2 μL/unilateral) decreased TSTQ significantly compared to the naive and naive + FSK groups. Moreover, there was a dose-dependent improvement after the long-term administration of FSK (15, 30, and 45 mg/kg) compared to the 6-OHDA-treated group [F_5,25_ = 0.13930; ANOVA (one-way); *p* < 0.01] (Figure 1b). A high dose of FSK (45 mg/kg) helps restore memory and raises the TSTQ against 6-OHDA, which causes PD in rats.

#### 2.1.2. FSK Restored Muscle Grip Strength in the Experimental Model of PD

**Figure 2:**
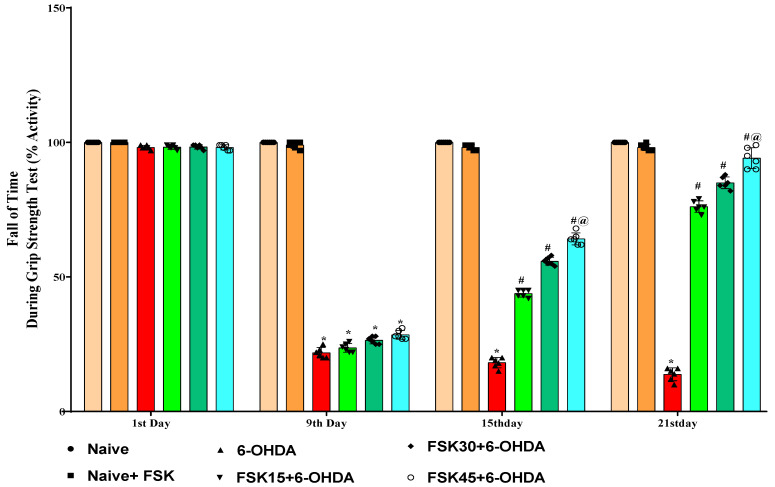
FSK mediated muscle grip restoration using the grip strength task in the experimental model of PD. *Data provided as mean ± SD (n = 6) followed by ANOVA (two-way) with post-hoc Bonferroni test.* * 6-OHDA (*p* < 0.01) compared with naive *and naive + FSK*; *# FSK15, FSK30, FSK45 (p <* 0.01*)* compared with *6-OHDA*; *#@ FSK45 (p < *0.01*)* compared with *FSK30, FSK15*.

On the first, ninth, fifteenth and twenty-first day of the procedure, the percentage of activity (fall-off time) on the grip strength apparatus was measured to evaluate the muscle strength of the rats. The naive and naive + FSK (45 mg/kg p.o.) groups showed no statistically significant differences. In contrast to the naive and naive + FSK groups, the chronic administration of 6-OHDA (8 μg/2 μL/unilateral) caused a gradual decrease in grip strength as measured by the time required to hold a metal wire. Moreover, the chronic administration of FSK at doses of 15, 30 and 45 mg/kg revealed a dose-dependent grip strength improvement in comparison to the 6-OHDA-treated group [F_15,90_ = 1361.76; ANOVA (two-way); *p* < 0.01] (Figure 2). In rats with 6-OHDA-induced PD, treatment with a high dose of FSK (45 mg/kg) both lengthens the time needed to grasp a metal wire and makes restoring grip strength more effective.

#### 2.1.3. FSK Improved Memory Retention in the Experimental Model of PD

**Figure 3:**
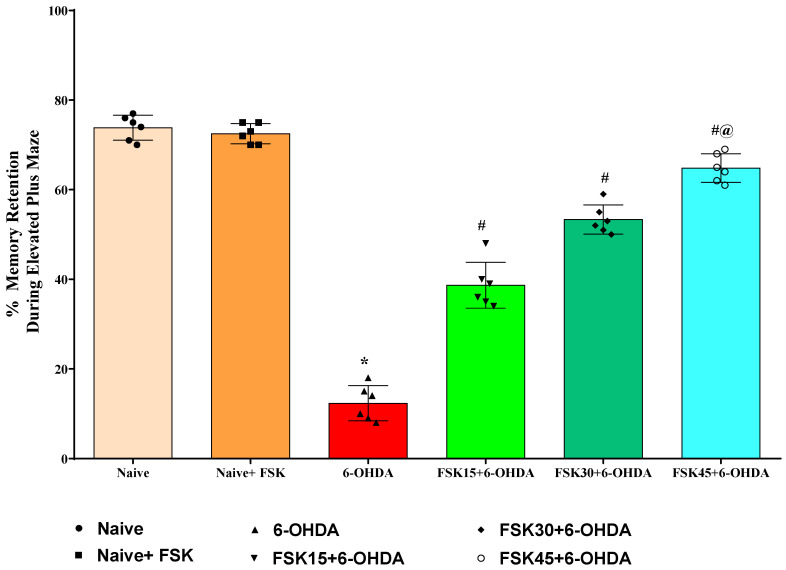
FSK improved memory retention using an elevated plus maze (EPM) in the experimental model of PD. *Data provided as mean ± SD (n = *6*) followed by ANOVA (one-way) with post-hoc Tukey’s test.* * 6-OHDA (*p* < 0.01) compared with naive *and naive + FSK*; *# FSK15, FSK30, FSK45 (p < *0.01*)* compared with *6-OHDA*; *#@ FSK45 (p < *0.01*)* compared with *FSK30, FSK15*.

The transfer latency (TL) task was used to test learning and memory in rats on the twentieth, twenty-first and twenty-second days of the protocol schedule. This study found no statistically significant differences between the naive and naive + FSK groups. In contrast to the naive and naive + FSK groups, however, the chronic injection of 6-OHDA (8 μg/2 μL/unilateral) significantly reduced the TL. In addition, chronic FSK (15, 30 and 45 mg/kg) showed a dose-dependent improvement over 6-OHDA [F_5,25_ = 2.119; ANOVA (one-way); *p* < 0.01] (Figure 3). The administration of a high dose of FSK (45 mg/kg) demonstrates a significant improvement in memory restoration and increases the TL in rats exposed to 6-OHDA, which causes PD.

#### 2.1.4. FSK Restored Neuromuscular Coordination in the Experimental Model of PD

**Figure 4:**
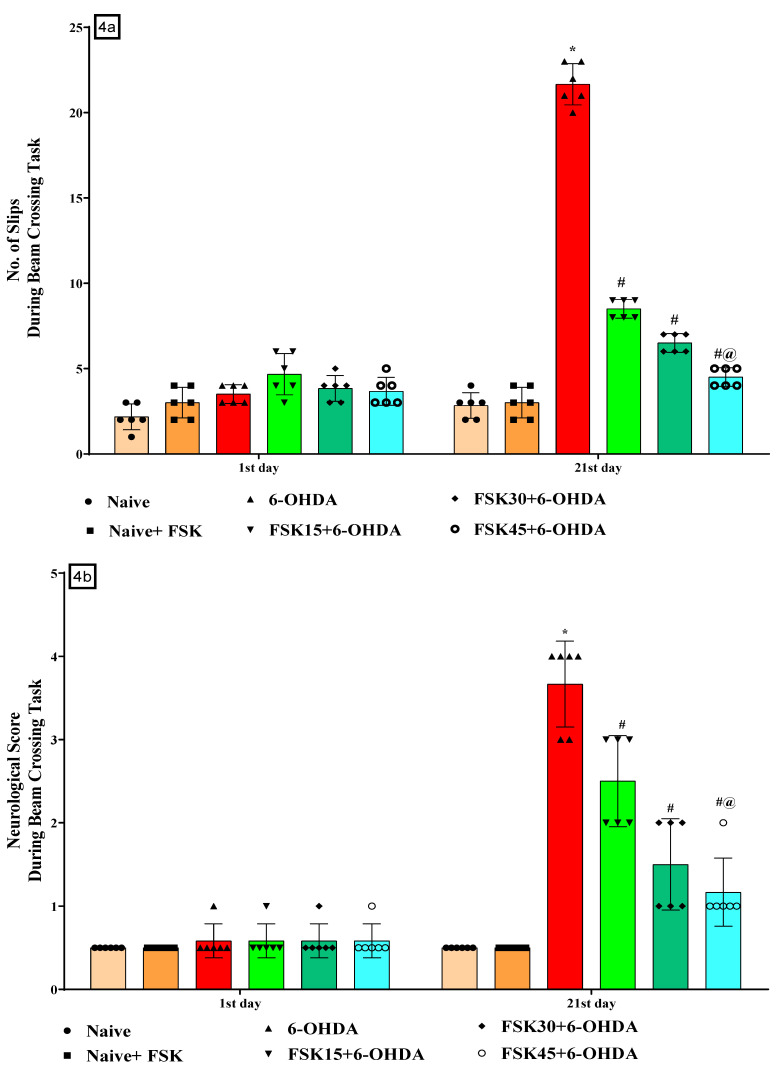
(**a**) FSK reduced the number of slips using the beam crossing task (BCT) in the experimental model of PD. *Data provided as mean ± SD (n =* 6*) followed by ANOVA (two-way) with post-hoc Bonferroni test.* * 6-OHDA (*p* < 0.01) compared with naive *and naive + FSK*; *# FSK15, FSK30, FSK45 (p < *0.01*)* compared with *6-OHDA*; *#@ FSK45 (p < *0.01*)* compared with *FSK30, FSK15*. (**b**) FSK reduced the neurological score using the beam crossing task (BCT) in the experimental model of PD. *Data provided as mean ± SD (n = *6*) followed by ANOVA (two-way) with post-hoc Bonferroni test.* * 6-OHDA (*p* < 0.01) compared with naive *and naive + FSK*; *# FSK15, FSK30, FSK45 (p < *0.01*)* compared with *6-OHDA*; *#@ FSK45 (p < *0.01*)* compared with *FSK30, FSK15*.

The number of slips on the NBW and neurological scores were recorded on days 1 and 21 of the protocol to assess the rats’ mobility impairment. The naive and naive + FSK (45 mg/kg p.o.) groups were compared, and there was no statistically significant difference between them. However, compared to the naive and naive + FSK groups, there was a gradual rise in the number of slips after the chronic administration of 6-OHDA (8 μg/2 μL/Unilateral). Moreover, the chronic administration of FSK at doses of 15, 30 and 45 mg/kg compared to the 6-OHDA-treated group resulted in a substantial reduction in the number of slips and an improvement in walking balance [F_5,30_ = 174.89; ANOVA (two-way); *p* < 0.01] (Figure 4a) and a dose-dependent reduction in the neurological score [F_5,30_ = 54.52; ANOVA (two-way); *p* < 0.01] (Figure 4b). A high dose of FSK (45 mg/kg) more effectively reduces the number of falls and improves the impairment and neurological score for beam walking in rats with 6-OHDA-induced PD.

#### 2.1.5. FSK Improved Locomotion in the Experimental Model of PD

**Figure 5:**
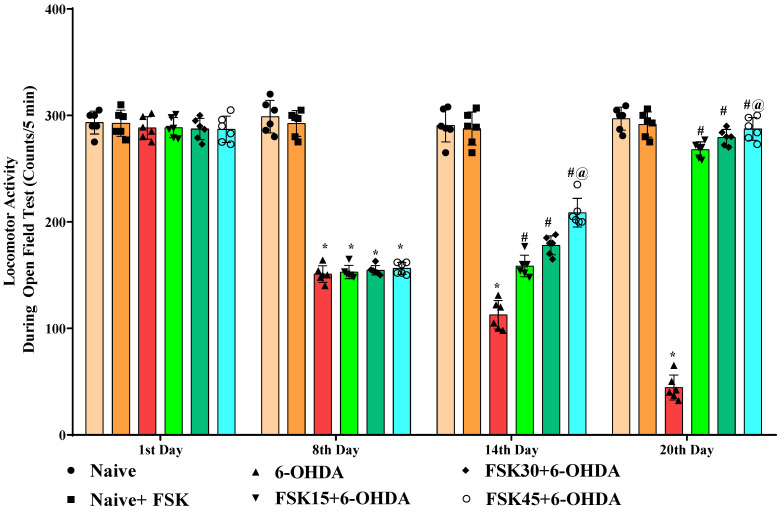
FSK improved locomotion using the open-field test in the experimental model of PD. *Data provided as mean ± SD (n = *6*) followed by ANOVA (two-way) with post-hoc Bonferroni test.* * 6-OHDA (*p* < 0.01) compared with naive *and naive + FSK*. *# FSK15, FSK30, FSK45 (p < *0.01*)* compared with *6-OHDA*; *#@ FSK45 (p < *0.01*)* compared with *FSK30, FSK15*.

Rats’ locomotor activity (LA) was evaluated using open field apparatus on the protocol schedule’s first, eighth, fourteenth and twentieth days. The naive and naive + FSK groups displayed no statistically significant differences. Conversely, compared to the naive and naive + FSK groups, the chronic treatment of 6-OHDA (8 μg/2 μL/unilateral) significantly reduced locomotor movement in rats. Furthermore, there was a substantial improvement in locomotion demonstrated following the chronic administration of FSK (15, 30 and 45 mg/kg) in a dose-dependent manner in comparison to the 6-OHDA treated group [F_15,90_ = 166.49; ANOVA (two-way); *p* < 0.01] (Figure 5). In rats, a high dose of FSK (45 mg/kg) improves and promotes locomotor movement against 6-OHDA-induced PD.

#### 2.1.6. FSK Restored Voluntary Movement in the Experimental Model of PD

**Figure 6:**
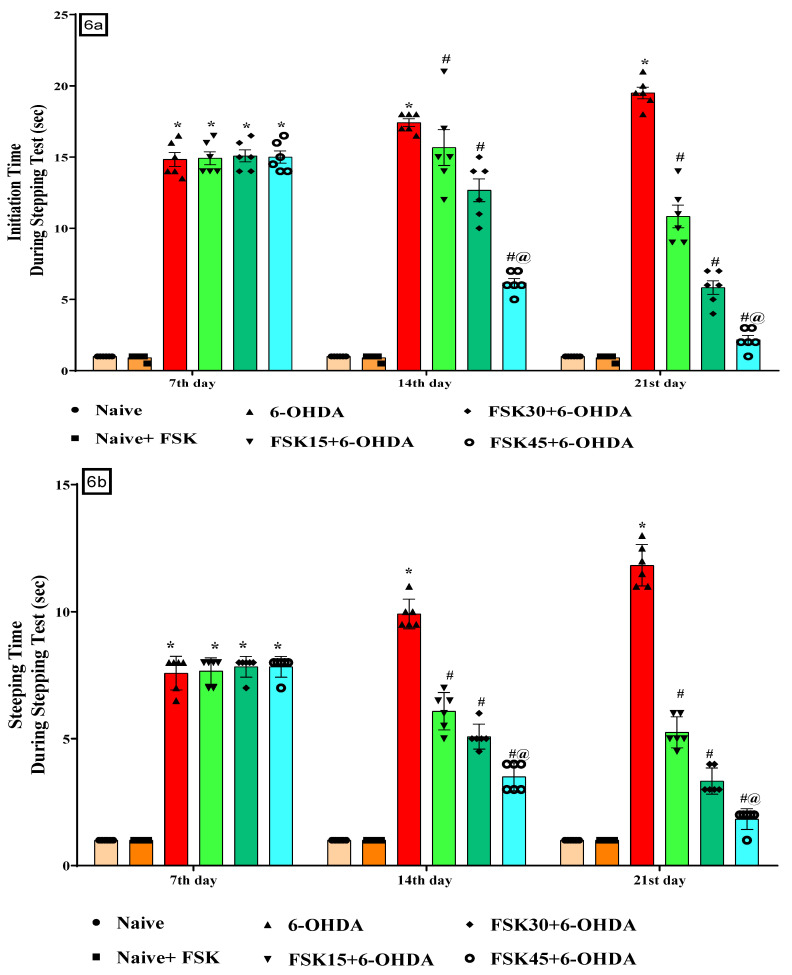

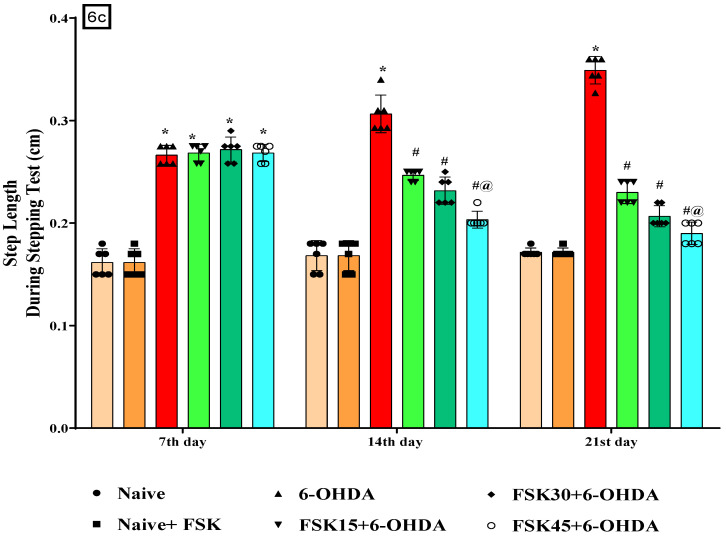
(**a**) FSK reduced initiation time using the stepping test in the experimental model of PD; (**b**) FSK reduced stepping time using the stepping test in the experimental model of PD; (**c**) FSK reduced step length using the stepping test in the experimental model of PD. *Data provided as mean ± SD (n* = 6*) followed by ANOVA (two-way) with post-hoc Bonferroni test.* * 6-OHDA (*p* < 0.01) compared with naive *and naive + FSK*; *# FSK15, FSK30, FSK45 (p < *0.01*)* compared with *6-OHDA*; *#@ FSK45 (p < *0.01*)* compared with *FSK30, FSK15*.

According to the protocol, akinesia on the stepping apparatus was performed on the seventh, fourteenth and twenty-first days to assess the impairment in voluntary movement. There was no statistically significant difference between the naive and naive + FSK (45 mg/kg p.o.) groups. Compared to the naive and naive + FSK groups, there was a steady reduction of voluntary movement after the chronic injection of 6-OHDA (8 μg/2 μL/unilateral). The chronic treatment of FSK at 15, 30 and 45 mg/kg decreased the initiation time [F_10,60_ = 53.07; ANOVA (two-way); *p* < 0.01] (Figure 6a), stepping time [F_10,60_ = 104.30; ANOVA (two-way); *p* < 0.01] (Figure 6b), and step length [F_10,60_ = 43.53; ANOVA (two-way); *p* < 0.01] (Figure 6c). A high dose of FSK (45 mg/kg) enhances and restores voluntary movement in rats suffering from 6-OHDA-induced PD.

#### 2.1.7. FSK Improved Motor Coordination in the Experimental Model of PD

**Figure 7:**
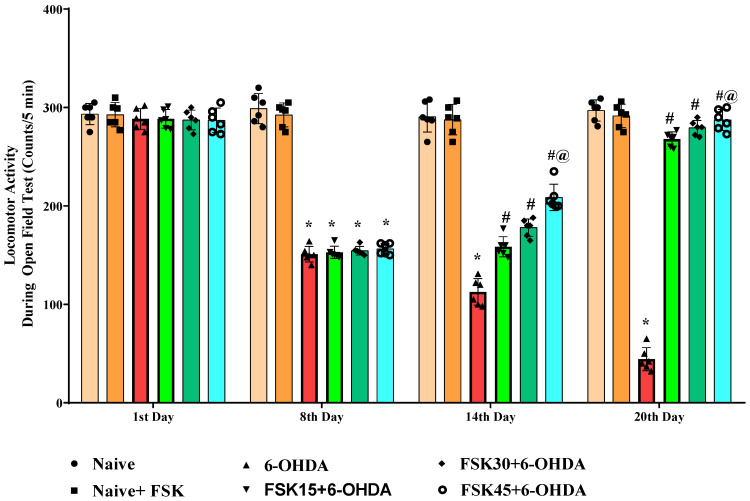
FSK improved motor coordination using the rotarod test in the experimental model of PD. *Data provided as mean ± SD (n = 6) followed by ANOVA (two-way) with post-hoc Bonferroni test.* * 6-OHDA (*p* < 0.01) compared with naive *and naive + FSK*; *# FSK15, FSK30, FSK45 (p < *0.01*)* compared with *6-OHDA*; *#@ FSK45 (p < *0.01*)* compared with *FSK30, FSK15*.

The rotarod task was used to test motor coordination in rats on the first, seventh, fourteenth and twenty-first days of the protocol schedule. The naive and naive + FSK groups showed no statistically significant differences. The rats’ motor coordination significantly declined after chronic 6-OHDA (8 μg/2 μL/unilateral) treatment. Moreover, there was a dose-dependent improvement in neuromuscular coordination after the long-term administration of FSK (15, 30 and 45 mg/kg) compared to the 6-OHDA-treated group [F_15,90_ = 347.89; ANOVA (two-way); *p* < 0.01] (Figure 7). When given to 6-OHDA-induced PD rats, a high dose of FSK (45 mg/kg) significantly improves coordination restoration and increases motor movement.

### 2.2. Biochemical Parameters

#### 2.2.1. FSK Increases the Myelin Basic Protein (MBP) Level in the Experimental Model of PD

**Figure 8:**
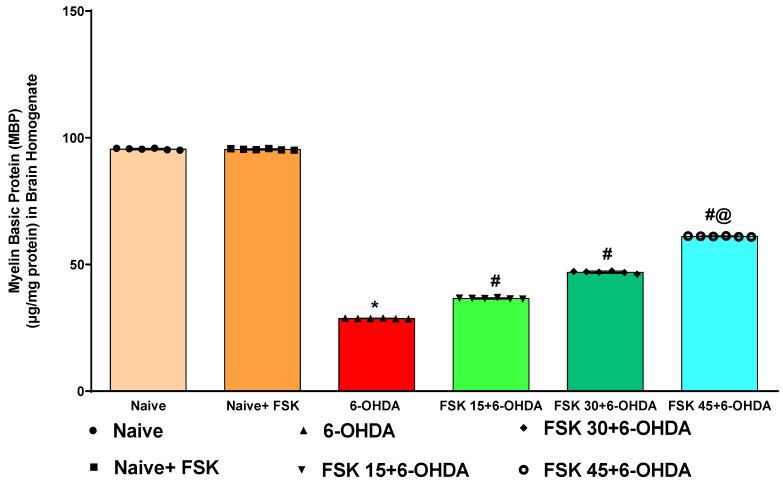
FSK increases myelin basic protein (MBP) levels in the experimental model of PD. *Data provided as mean ± SD (n = *6*) followed by ANOVA (one-way) with post-hoc Tukey’s test.* * 6-OHDA (*p* < 0.01) compared with naive *and naive + FSK*; *# FSK15, FSK30, FSK45 (p < *0.01*)* compared with *6-OHDA*; *#@ FSK45 (p < *0.01*)* compared with *FSK30, FSK15*.

Demyelination in the rat brain homogenate was estimated at the end of the protocol schedule. The naive and naive + FSK (45 mg/kg p.o.) groups showed no statistically significant differences. Compared to the naive and naive + FSK groups, the prolonged administration of 6-OHDA (8 μg/2 μL/unilateral) resulted in a gradual loss of MBP and increased demyelinated neurons. MBP was significantly improved in a dose-dependent manner following the continuous administration of FSK at 15, 30 and 45 mg/kg compared to the 6-OHDA-treated group [F_5,25_ = 0.8830; ANOVA (one-way); *p* < 0.01] (Figure 8). A high dose of FSK (45 mg/kg) raises, recovers and controls the level of MBP in rats suffering from 6-OHDA-induced Parkinsonism.

#### 2.2.2. FSK Mediated the Restoration of Cellular and Molecular Alterations in the Experimental Model of PD

##### FSK Restored the ETC-Complexes (I, II, V) Mitochondrial Enzyme Levels in the Experimental Model of PD

**Figure 9:**
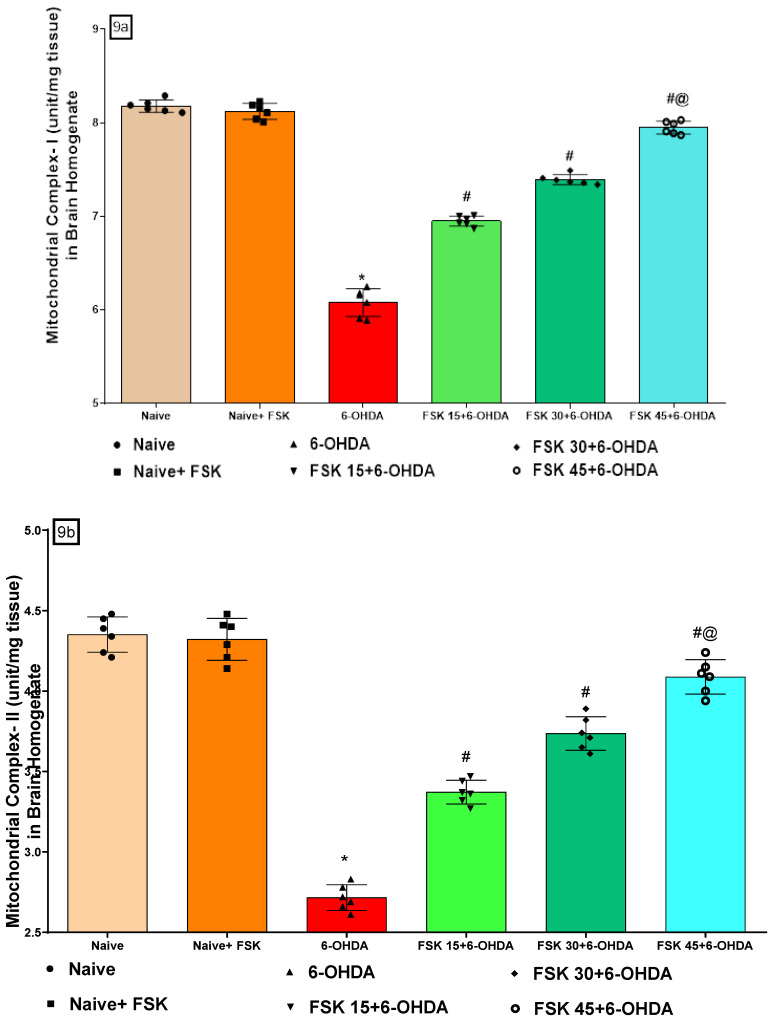

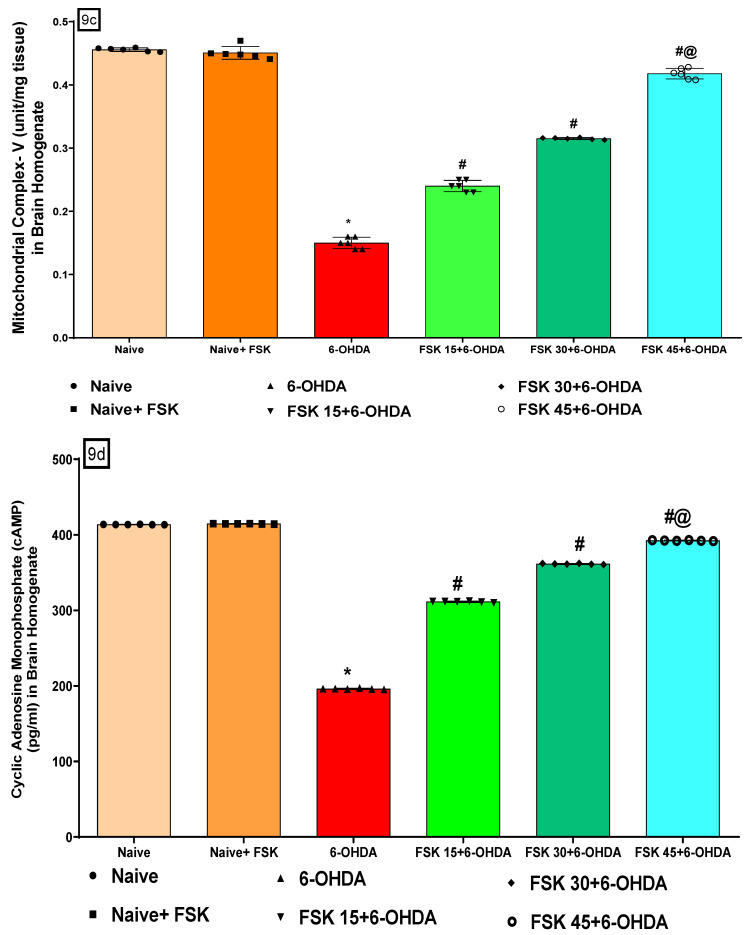

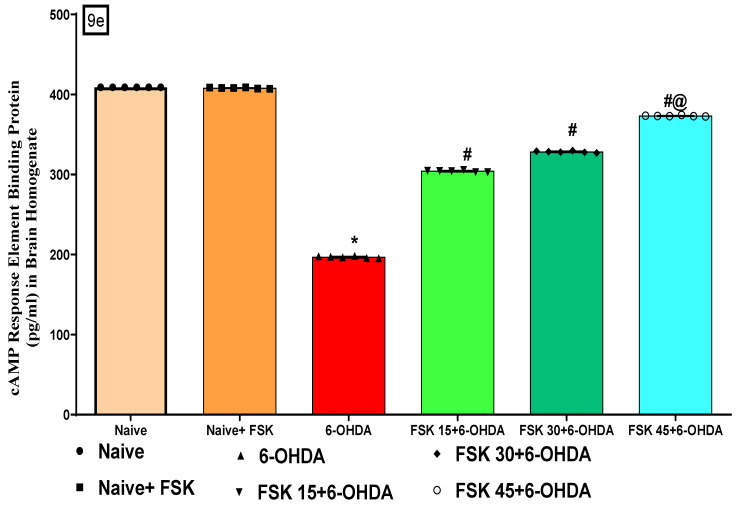
(**a**–**c**) FSK restored ETC-complexes (I, II, V) enzyme levels in the experimental model of PD. *Data provided as mean ± SD (n = *6*) followed by ANOVA (one-way) with post-hoc Tukey’s test.* * 6-OHDA (*p* < 0.01) compared with naive *and naive + FSK*; *# FSK15, FSK30, FSK45 (p < *0.01*)* compared with *6-OHDA*; *#@ FSK45 (p < *0.01*)* compared with *FSK30, FSK15*. (**d**,**e**) FSK improved cAMP and CREB protein levels in the experimental model of PD. *Data provided as mean ± SD (n = *6*) followed by ANOVA (one-way) with post-hoc Tukey’s test.* * 6-OHDA (*p* < 0.01) compared with naive *and naive + FSK # FSK15, FSK30, FSK45 (p < *0.01*)* compared with *6-OHDA*; *#@ FSK45 (p < *0.01*)* compared with *FSK30, FSK15*.

Mitochondrial enzyme complex I (NADPH dehydrogenase), complex II (succinate dehydrogenase (SDH)), and complex V (ATP) activity were measured in the rat brain homogenate at the end of the experimental schedule. No significant differences were observed between the naive and naive + FSK (45 mg/kg p.o.) groups. Nonetheless, compared to the naive and naive + FSK groups, rat complex I, II and V activity was significantly reduced after the chronic injection of 6-OHDA (8 μg/2 μL/unilateral). Moreover, mitochondrial complex I [F_5,25_ = 1.224; ANOVA (one-way); *p* < 0.01] (Figure 9a), complex II [F_5,25_ = 0.7712; ANOVA (one-way); *p* < 0.01] (Figure 9b) and complex V [F_5,25_ = 0.9201; ANOVA (one-way); *p* < 0.01] (Figure 9c) activity improved dose-dependently following chronic FSK (15, 30 and 45 mg/kg) treatment compared to the 6-OHDA treated group. A high FSK (45 mg/kg) dosage improves complex I, II and V activity against 6-OHDA-induced PD in rats.

##### FSK Improved cAMP and CREB Protein Levels in the Experimental Model of PD

At the end of the protocol schedule, molecular markers (cAMP and CREB) were measured in the rats’ brain homogenate. The naive and naive + FSK (45 mg/kg p.o.) groups showed no statistically significant differences. In contrast to the naive and naive + FSK groups, the amount of cAMP and CREB proteins gradually decreased following the continuous injection of 6-OHDA (8 μg/2 μL/unilateral). Furthermore, the chronic administration of FSK at doses of 15, 30 and 45 mg/kg compared to the 6-OHDA-treated group showed a significant increase in cAMP [F_5,25_ = 1.660; ANOVA (one-way); *p* < 0.01 (Figure 9d) and CREB [F_5,25_ = 1.546; ANOVA (one-way); *p* < 0.01] (Figure 9e) levels in a dose-dependent manner. In rats with 6-OHDA-induced PD, a high dose of FSK (45 mg/kg) demonstrates a significant improvement in restoring the levels of cAMP and CREB.

#### 2.2.3. FSK Modulated Inflammatory Cytokines in the Experimental Model of PD

**Figure 10:**
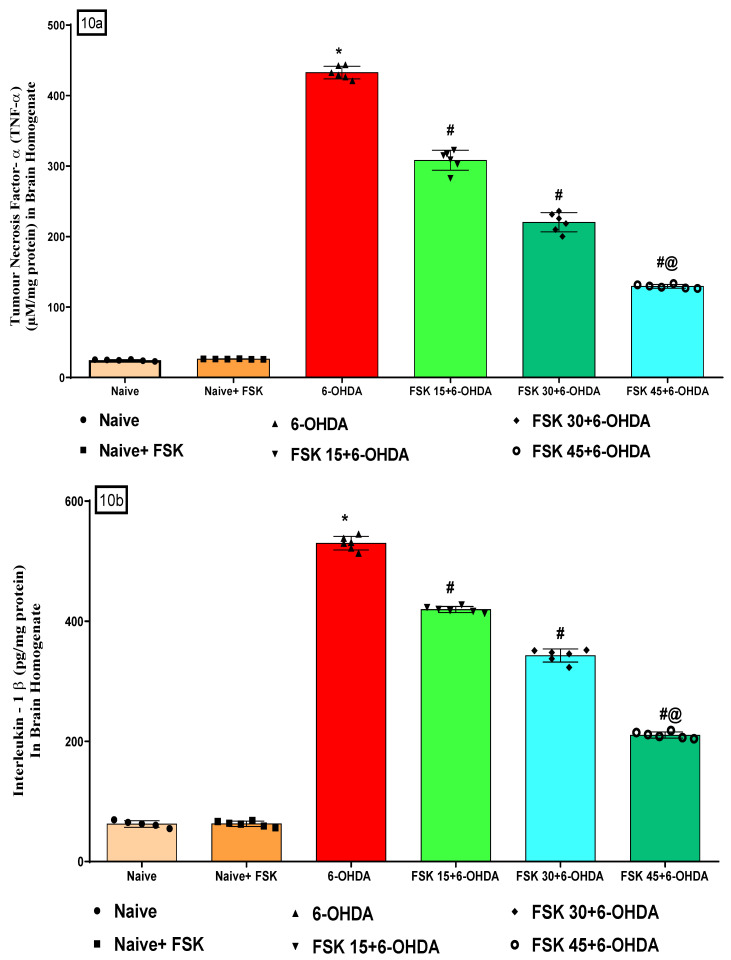

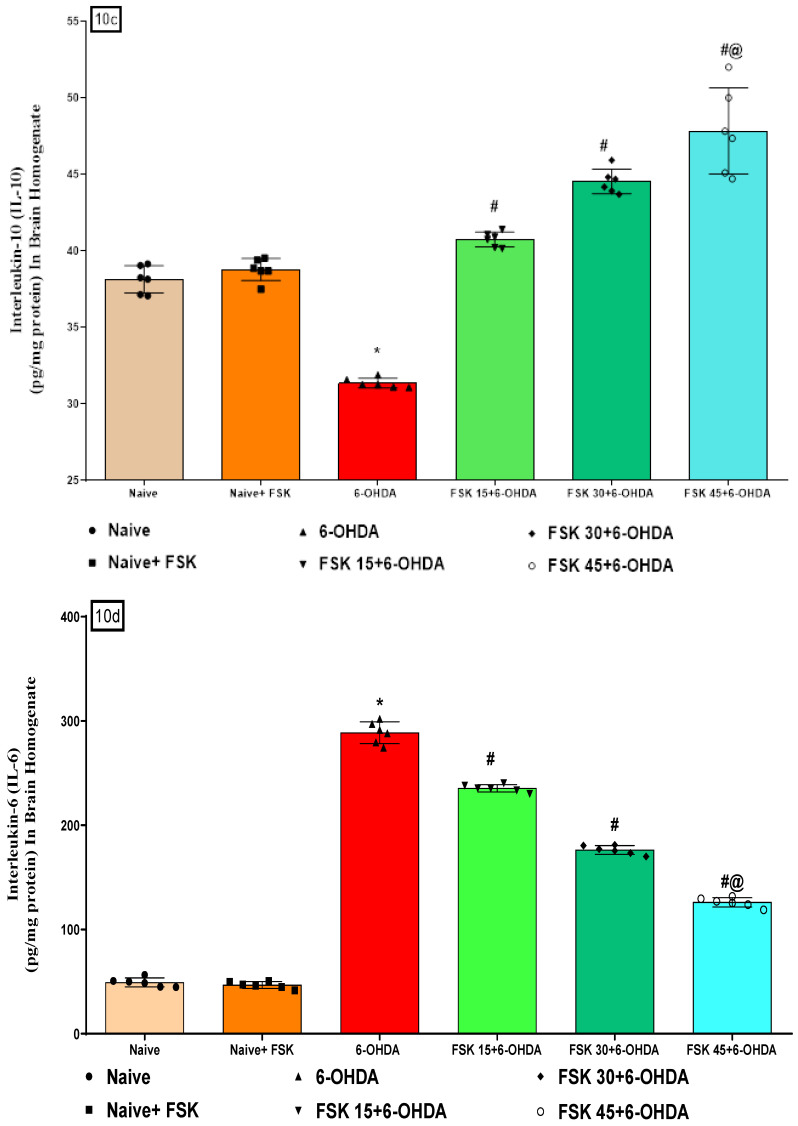
(**a**–**d**) FSK modulated inflammatory cytokines in the experimental model of PD. *Data provided as mean ± SD (n = *6*) followed by ANOVA (one-way) with post-hoc Tukey’s test*. * 6-OHDA (*p* < 0.01) compared with naive *and naive + FSK; # FSK15, FSK30, FSK45 (p < *0.01*)* compared with *6-OHDA*; *#@ FSK45 (p < *0.01) compared with *FSK30, FSK15*.

At the end of the treatment, inflammatory cytokines (TNF-α, IL-1β, IL-6, IL-10) were measured in the rats’ brain homogenate. Naive and Naive + FSK (45 mg/kg p.o.) showed no statistically significant differences. Compared to the naive and naive + FSK groups, there was a steady increase in TNF-α, IL-1β and IL-6 levels and a decrease in IL-10 levels after the chronic administration of 6-OHDA (8 μg/2 μL/unilateral). The chronic treatment of FSK at 15, 30 and 45 mg/kg decreased TNF-α [F_5,25_ = 0.1271; ANOVA (one-way); *p* < 0.01] (Figure 10a), IL-1β [F_5,25_ = 2.388; ANOVA (one-way); *p* < 0.01 (Figure 10b), IL 6 [F_5,25_= 8.503; ANOVA (one-way); *p* < 0.01] (Figure 10d). On the other hand, it dose-dependently raised IL-10 [F_5,25_ = 2.239; ANOVA (one-way); *p* < 0.01] (Figure 10c). In rats with 6-OHDA-induced PD, a high dose of FSK (45 mg/kg) demonstrates a significant improvement in recovering inflammatory markers.

#### 2.2.4. FSK Restored Neurotransmitters in the Experimental Model of PD

**Figure 11:**
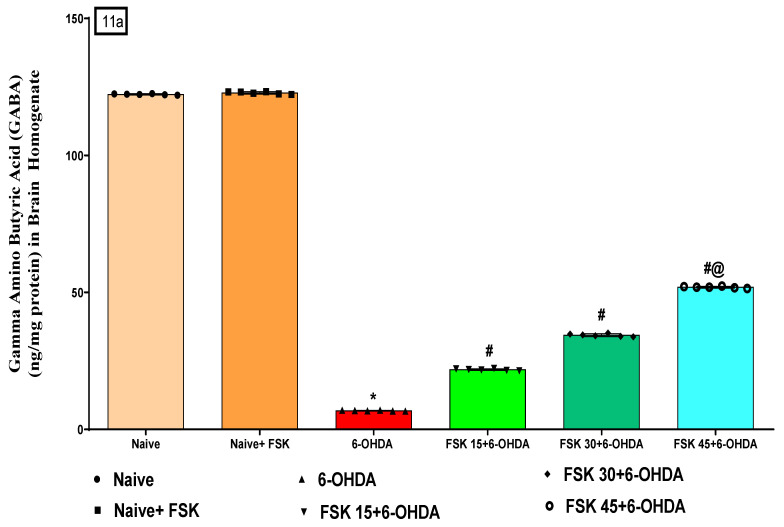

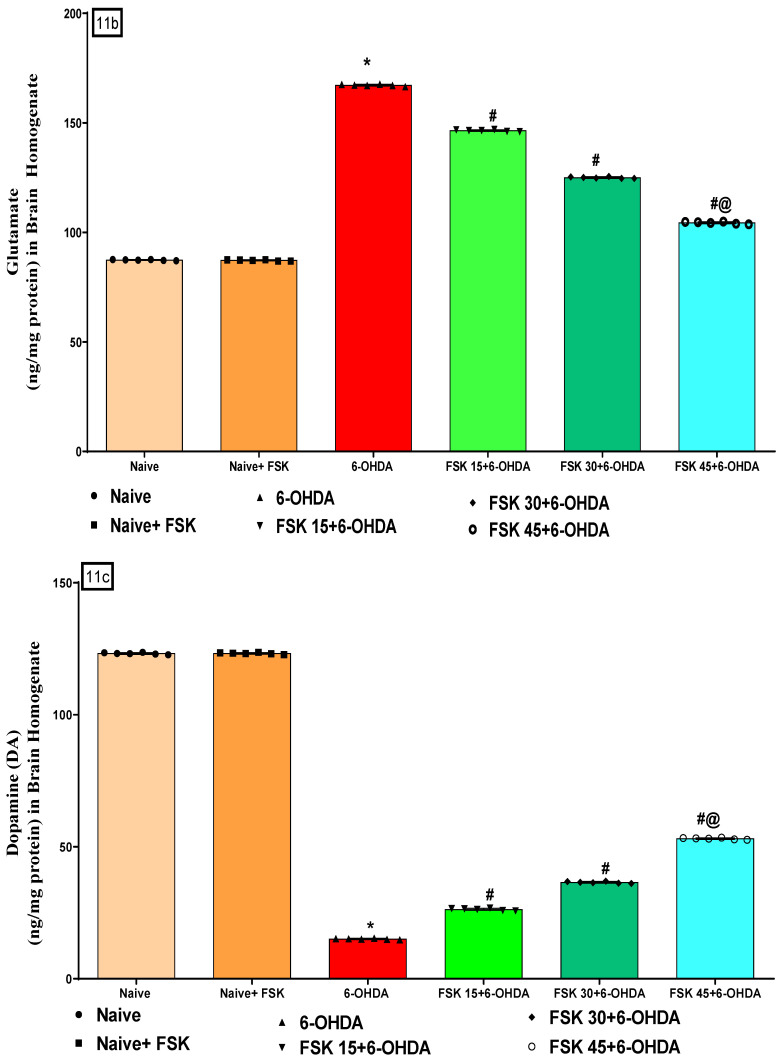

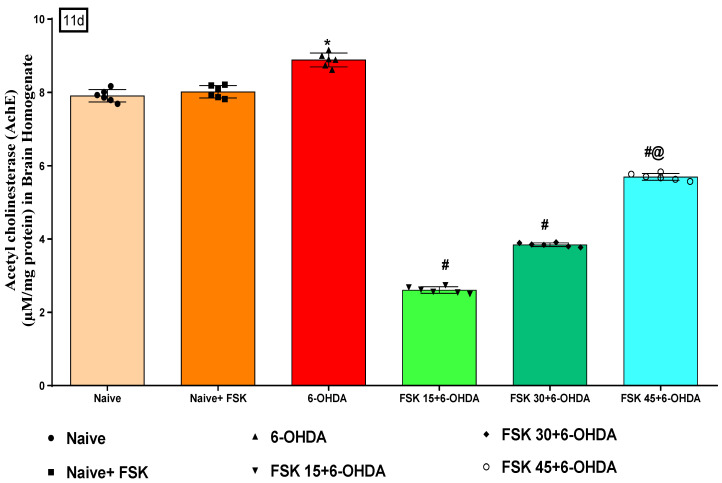
**(a**–**d)** FSK restored neurotransmitters in the experimental model of PD. *Data provided as mean ± SD (n* = 6*) followed by ANOVA (one-way) with post-hoc Tukey’s test.* * 6-OHDA (*p* < 0.01) compared with naive and *naive + FSK; # FSK15, FSK30, FSK45 (p < *0.01*)* compared with *6-OHDA*; *#@ FSK45 (p < *0.01*)* compared with *FSK30, FSK15*.

Neurotransmitter levels (DA, glutamate, GABA and Ach) were measured in rat brain homogenates at the end of the experimental schedule. The naive and naive + FSK (45 mg/kg p.o.) groups showed no statistically significant difference. However, GABA and DA levels gradually decreased. In addition, the prolonged injection of 6-OHDA (8 μg/2 μL/unilateral) resulted in elevated Ach and glutamate levels in demyelinated neurons compared to the naive and naive + FSK groups. Moreover, compared to the 6-OHDA-treated group, the chronic administration of FSK at doses of 15, 30 and 45 mg/kg resulted in substantial increases in GABA [F_5,25_ = 0.2122; ANOVA (one-way); *p* < 0.01] (Figure 11a) [F_5,25_ = 0.1.322; ANOVA (one-way); *p* < 0.01] (Figure 11c). On the other hand, reducing Ach [F_5,25_ = 0.4141; ANOVA (one-way); *p* < 0.01] (Figure 11d) levels and reduces glutamate levels in a dose-dependent manner [F_5,25_ = 0.7503; ANOVA (one-way); *p* < 0.01] (Figure 11d). FSK at a high dose (45 mg/kg) modulates the neurotransmitter level in 6-OHDA and induces PD in rats.

#### 2.2.5. FSK Restored Anti-Oxidant Levels in the Experimental Model of PD

**Figure 12:**
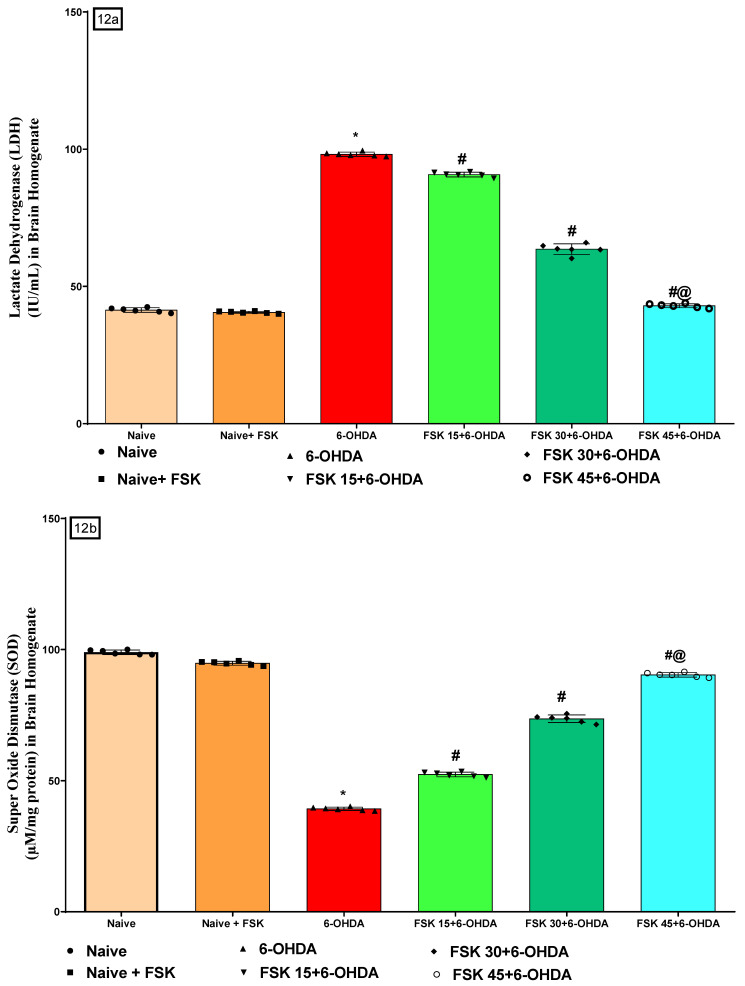

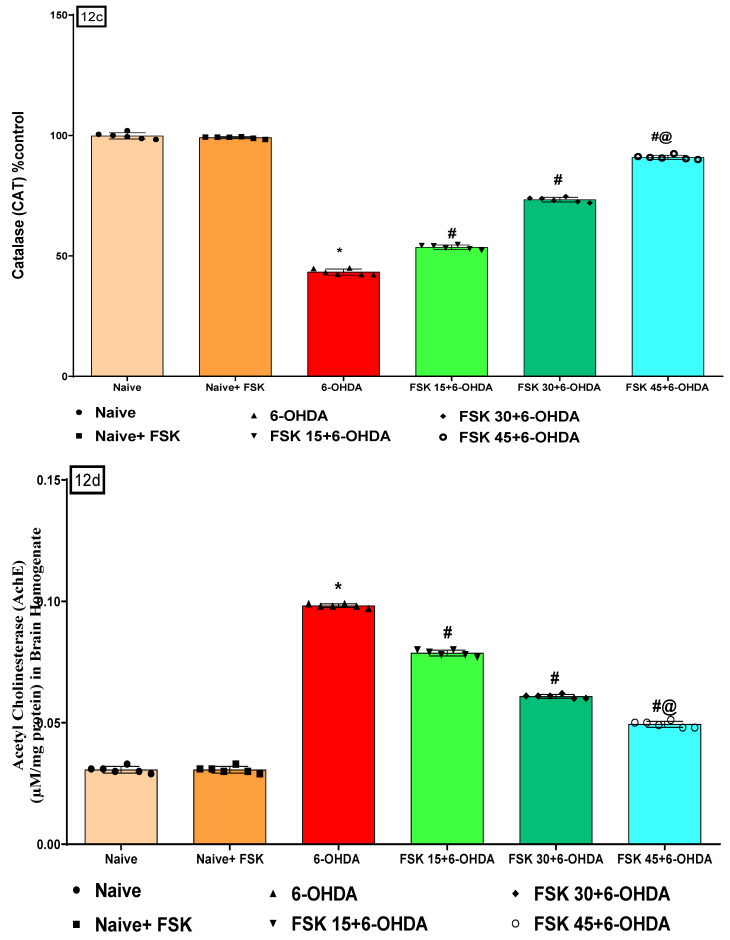

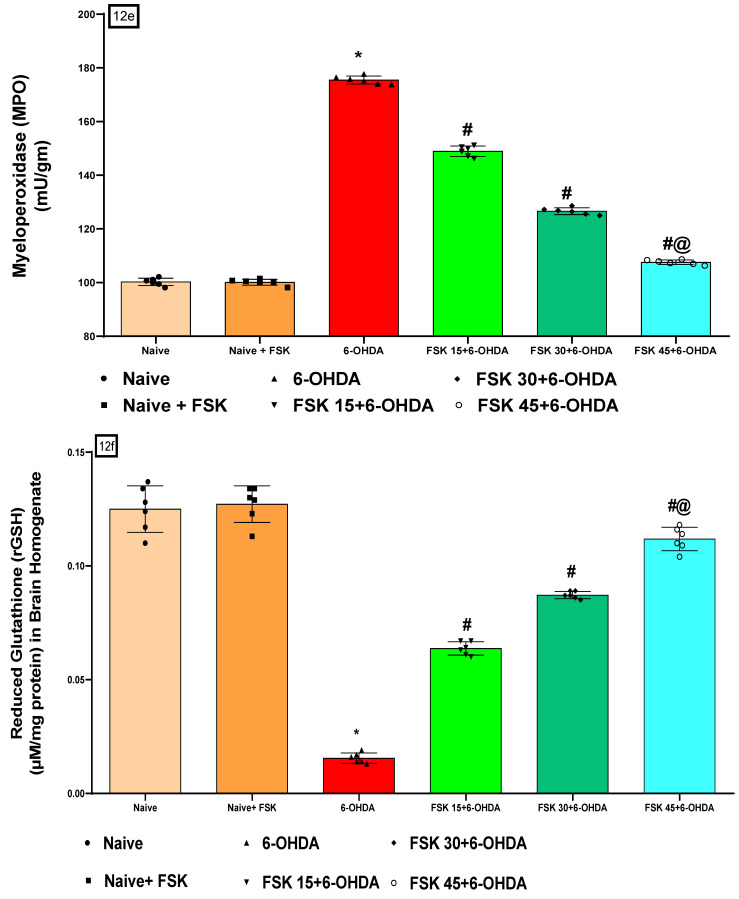

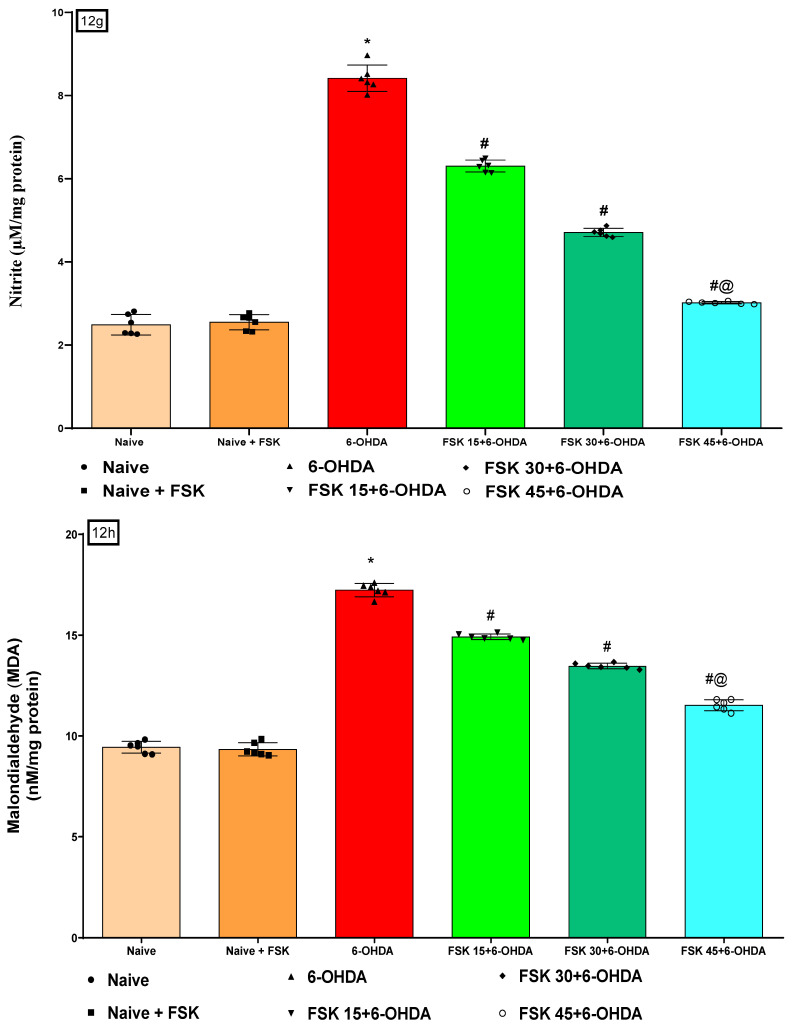

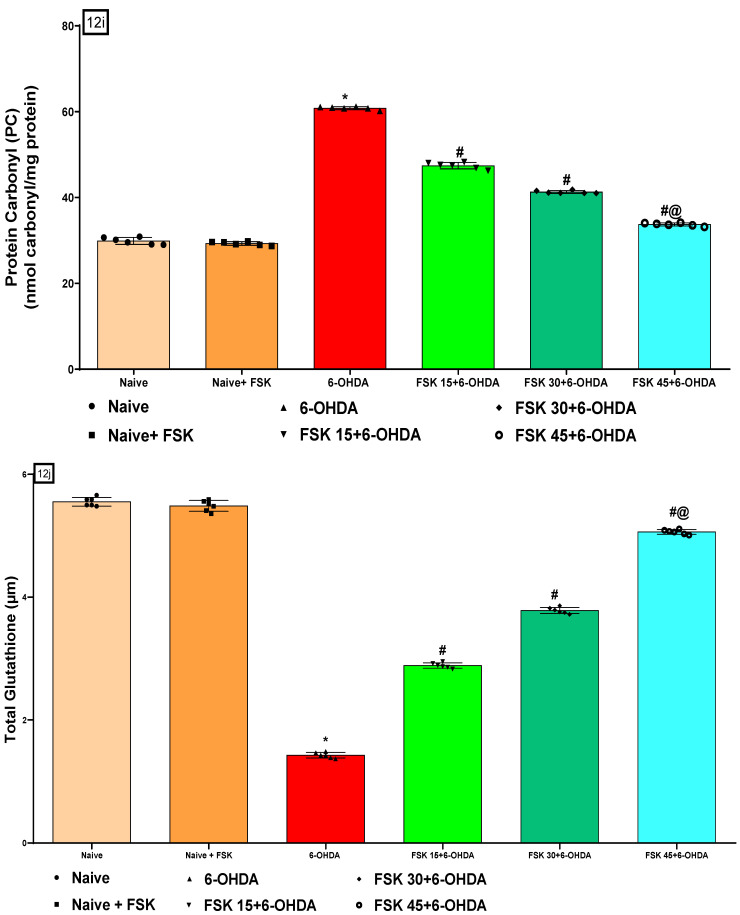

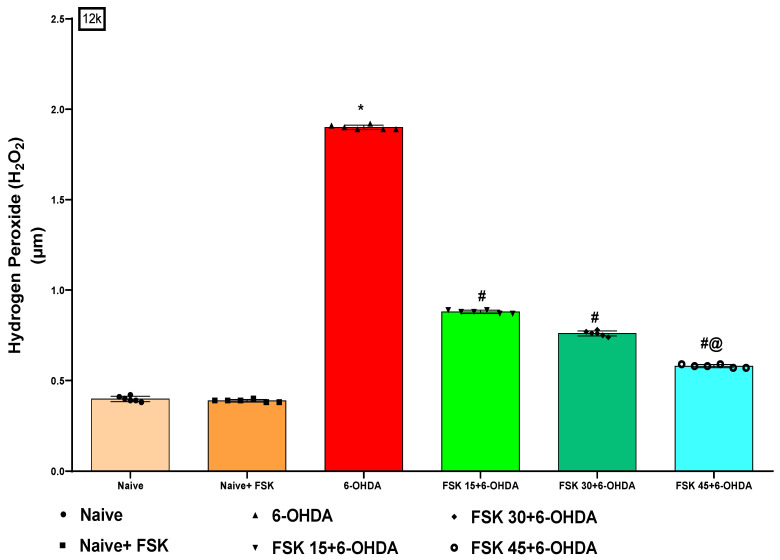
(**a**–**k**) FSK restored anti-oxidant levels in the experimental model of PD. *Data provided as mean ± SD (n =* 6) followed by ANOVA (one-way) with post-hoc Tukey’s test. * 6-OHDA (p < 0.01*) compared with naive and naive + FSK; # FSK15, FSK30, FSK45 (p < *0.01*) compared with 6-OHDA; #@ FSK45 (p < *0.01*) compared with FSK30, FSK15*.

In the rat brain homogenate, oxidative stress indicators, including LDH, SOD, CAT, AchE, MPO, GSH, nitrite, MDA, PC, total glutathione (GSH) and H_2_O_2_ were measured at the end of the protocol. The naive and naive + FSK (45 mg/kg p.o.) groups showed no statistically significant difference. On the other hand, there was a slow but gradual decline in SOD, CAT, GSH and total GSH levels. In contrast, the prolonged injection of 6-OHDA (8 μg/2 μL/Unilateral) resulted in elevated levels of LDH, AchE, MPO, nitrite, MDA, PC and H_2_O_2_. Furthermore, the chronic treatment of FSK at doses of 15, 30 and 45 mg/kg compared to the 6-OHDA-treated group resulted in a significant increase in SOD [F_5,25_ = 1.547; ANOVA (one-way); *p* < 0.01] (Figure 12b), CAT [F_5,25_ = 1.429; ANOVA (one-way); *p* < 0.01] (Figure 12c), GSH [F_5,25_ = 0.07114; ANOVA (one-way); *p* < 0.01] (Figure 12f) and total GSH [F_5,25_ = 4.594; ANOVA (one-way); *p* < 0.01] (Figure 12j) levels. Whereas, the levels of LDH [F_5,25_ = 5.484; ANOVA (one-way); *p* < 0.01] (Figure 12a), AchE [F_5,25_ = 0.7693; ANOVA (one-way); *p* < 0.01] (Figure 12d), MPO [F_5,25_ = 2.196; ANOVA (one-way); *p* < 0.01] (Figure 12e), nitrite [F_5,25_ = 3.836; ANOVA (one-way); *p* < 0.01] (Figure 12g), MDA [F_5,25_ = 747.5; ANOVA (one-way); *p* < 0.01] (Figure 12h), PC [F_5,25_ = 1.159; ANOVA (one-way); *p* < 0.01] (Figure 12i), as well as H_2_O_2_ [F_5,25_ = 2.486; ANOVA (one-way); *p* < 0.01] (Figure 12k) were decreased in a dose-dependent manner. FSK (45 mg/kg) restores and regulates the levels of oxidative markers that induce PD in rats.

#### 2.2.6. FSK Prevents Histopathological Alterations in a Striatal Brain Region in the Experimental Model of PD

**Figure 13:**
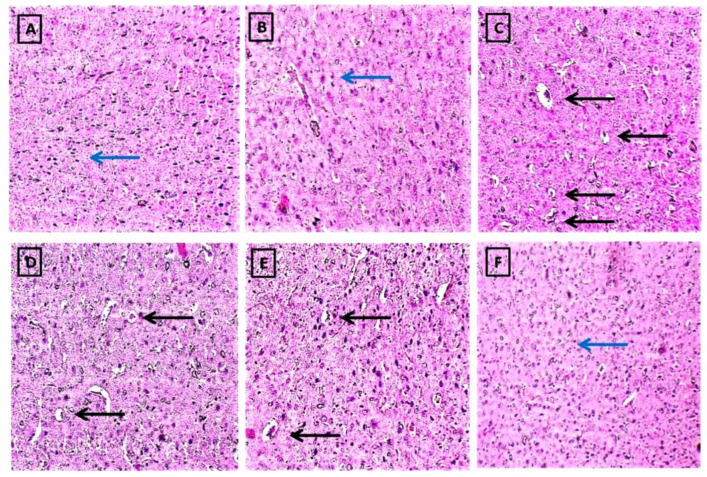
FSK prevents histopathological alterations in the striatal brain region in the experimental model of PD.

After the protocol schedule, histopathology was seen on the stained sectioned slides of the striatum region of the rat brains. The naive and naïve (13A) + FSK (45 mg/kg p.o.) (13B) groups showed no significant histopathological changes. However, after the continuous injection of 6-OHDA (8 μg/2 μL/unilateral) (13C), there were progressive increases in Lewy body formation, neurodegeneration and apoptosis compared to the naive and naive + FSK treated groups. Moreover, the continuous treatment of FSK at doses of 15, 30 and 45 mg/kg (13D,E,F) decreased the development and deposition of Lewy bodies in a dose-dependent manner compared to the 6-OHDA-treated group. FSK at high doses (45 mg/kg) reverses 6-OHDA-induced PD in rats by decreasing degeneration and apoptosis while restoring normal Lewy body development (Figure 13A–F).

Staining of the striatal brain sections with haematoxylin and eosin (H and E) reveals changes in neuronal cell density, dopaminergic neuron loss and the presence of Lewy bodies. Dopaminergic neuron loss causes neuronal degeneration and the formation of Lewy bodies in the rat brain. On the slides, the blue arrow represents neuronal density, while the black arrow represents the formation of Lewy bodies.

*(A) The diagram depicts the naive group, which has a high density of neuronal cells.*

*(B) The diagram depicts the FSK per se group, and there was no significant difference in neuronal cell density between the naive and naive + FSK groups.*

*(C) Diagram of the 6-OHDA group in which Lewy bodies are formed with low neuronal density.*

*(D–F) The diagram compares FSK 15, FSK 30 and FSK 45 to the 6-OHDA-treated group, which had significantly less Lewy body deposition and neuronal degeneration.*

*(F) The FSK 45 group had a higher restoration frequency of neurons with a higher neuronal density than the FSK 15 and 30 groups.*

## 3. Discussion

6-OHDA is a neurotoxin that inhibits complexes I and IV, causing mitochondrial dysfunction and, eventually, the death of catecholaminergic neurons (DA, adrenaline and noradrenaline) [35]. It does not penetrate the blood-brain barrier and is injected directly into the brain using stereotaxic surgery. An increasing body of research has shown that 6-OHDA induces motor impairment [21].

MWM and EPM were employed in this study to investigate memory and cognition-related behavioural activities. Rats given 6-OHDA had considerable learning and memory impairment compared to the naive and FSK-treated groups. In rats, 6-OHDA induced abnormal motor and behavioural patterns such as bradykinesia, muscular weakness, postural imbalance, dyskinesia and rigidity. PD rats with impaired learning and memory due to 6-OHDA administration exhibited aberrant motor test performance on the EPM, rotarod, stepping test, MWM, beam crossing and grip strength. According to this research, the administration of 6-OHDA to rats led to a wide range of neurobehavioral abnormalities, such as cognitive impairment, psychosocial problems and motor impairments. FSK decreased TL while increasing time spent in the target quadrant, grip strength and motor activities in a dose-dependent manner. Several neurotransmitter processes, including dopaminergic, glutamatergic, cholinergic and GABAergic, exhibited significant neurochemical alterations after the unilateral injection of 6-OHDA into the striatum of rats [36]. The experimental validation of 6-OHDA lesion model is influenced by biochemical changes (DA depletion, neurotransmitter changes), nigrostriatal pathological changes (SNpc cells loss > VTA), and forelimb akinesia, as well as mitochondrial dysfunction, oxidative stress and inflammation [37].

DA neurodegeneration in the SN and striatal axonal loss resulted in DA deficiency in basal ganglia (BG) region [38]. A disruption in the function of the mitochondrial respiratory chain, which also increases free radical production and lowers ATP, impedes oxidative phosphorylation [39].

The level of cAMP decreases when the mitochondrial respiratory chain is disturbed, resulting in the loss of ATP [40]. Adenylcyclase is stimulated as neurotransmitters bind to GPCRs, producing cyclic AMP (cAMP) [41]. Additionally, the cAMP/PKA signalling pathway aids in developing long-term memories [42]. As a direct activator of adenylate cyclase (AC), FSK protects neurons from mitochondrial dysfunction by increasing cAMP, activating PKA and CREB. There is evidence that FSK increases AC activity in the hippocampus, prefrontal cortex and striatum regions [43].

CREB is a transcription factor activated by cAMP/PKA in learning and memory formations [44]. Acetylcholine esterase, often known as AChE, is the enzyme in people with Parkinson’s disease that is responsible for maintaining healthy cholinergic function [45]. In the current experiment, FSK could minimise the amount of AChE observed in the PD brain.

Direct SN lesions produce DAergic neuron degeneration and tyrosine-hydroxylase (TH) deficiency in striatal neuron terminals. Additional respiratory inhibition and oxidative stress are caused by direct interactions with complexes I and IV of the mitochondrial respiratory chain. Consequently, oxidative stress and mitochondrial respiratory failure are the hallmarks of dopaminergic degeneration [46]. Furthermore, reduced levels of free radical-scavenging enzymes (GPx and CAT) and higher levels of oxidative stress were also observed in PD conditions [47,48]. FSK administration resulted in a considerable increase in the antioxidant enzymes SOD, CAT and total GSH, as well as a significant decrease in the amount of the oxidised free radical identified as H_2_O_2_. 6-OHDA inhibits the mitochondrial respiratory chain complexes I and IV, which are responsible for the formation of free radicals [49]. Our studies also significantly restored complex I, II and V activities after long-term FSK treatment.

People with Parkinson’s disease have been reported to have increased levels of C-reactive protein (CRP) and inflammatory cytokines [50]. This is because activated glial cells cause neurotoxicity by releasing pro-inflammatory cytokines, including TNF- and IL-6. In 6-OHDA-treated rat brains, FSK therapy dramatically decreased inflammatory cytokines while increasing enzymatic LDH and MDA levels.

Cell death in the SNpc area of the brain is caused by the generation of Lewy bodies. H and E-stained brain slices revealed alterations in neuronal cell density, dopaminergic neuron loss and the presence of Lewy bodies [36]. Neuronal degeneration and the development of Lewy bodies are caused by the loss of dopaminergic neurons after 6-OHDA exposure. According to this study, FSK reduces Lewy body-induced neuronal cell degeneration, DAergic neuron repair and cell death.

This study’s findings show that, compared to naive controls, prolonged treatment with naive + FSK had no effect on behavioural or biochemical tests in rats. FSK at 15, 30 and 45 mg/kg p.o. treated rats performed better than 6-OHDA-treated rats on a variety of behavioural and biochemical markers. FSK-protected neurons from 6-OHDA-mediated neurotoxicity were prevented by striatal histopathological alterations. In addition, stimulating the AC was found to be a more effective method for minimising the neurotoxicity produced by 6-OHDA. The cAMP/PKA/CREB pathway may play a role in the neuroprotective and regenerative effects of AC activation. However, research has revealed that CREB levels decline significantly as Parkinson’s disease progresses. Based on these findings, we conclude that FSK, a direct AC activator, enhances the neuroprotective and mitochondrial complex I, II and V restorative activities of cAMP-mediated CREB.

Based on our findings, we suggest that FSK could be utilised to treat symptoms similar to Parkinson’s disease. The fundamental technique, however, must be validated through adenylcyclase gene research employing knock-in and knock-out procedures. These findings will mostly be supported by cellular marker tests such as Western blot analysis and immunohistochemistry. According to our findings, conventional drugs are more successful when combined with FSK and could be employed in Parkinson’s disease therapy. We also urge more research to better understand the connection between conventional drugs and PD therapies.

There are limitations to this study. The current findings are preliminary work to reinforce future investigation about the role of FSK in the progression of Parkinson’s disease. The small number of animals in each group after conducting numerous behavioural and neurochemical parameters is another concern, and further research must involve a larger number of animals in each group. In the current study, a sham control group was taken as a naive control. Furthermore, the precise location of the cannula implantation must be verified with gross pathological examinations using the whole brain and brain sectioning. The correct location of the cannula should have been confirmed by staining of the striatum; however, the behavioural changes (LA, fall-off time, rotation) suggest that the cannula are most likely positioned in the correct area as all of the 6-OHDA group display changes in these parameters on days 7–8 which are reversed by FSK treatment.

## 4. Materials and Methods

### 4.1. Experimental Animals

The Rajendra Institute of Technology and Sciences in Sirsa, Haryana, India, provided 36 male Wistar rats (22 weeks old; 220–250 g) for the experiment. They were housed in polyacrylic cages with soft bedding and a wire mesh top for two weeks to let them become used to the laboratory environment. A standard environment was maintained, with a temperature of 22 ± 2 °C, a humidity of 65 to 70%, a 12-h light-dark cycle and unlimited access to food and water. The experimental protocol was approved by the Institutional Animal Ethics Committee (IAEC) (RITS/IAEC/2014/03/06) as per the guidelines of the Committee for the purpose of control and supervision of experiments on animals (CPCSEA), Government of India (888/PO/Re/S/05/CPCSEA).

### 4.2. Drugs and Chemicals

Sigma-Aldrich was contacted for the acquisition of 6-OHDA (St. Louis, MO, USA). This batch of FSK came from the Indian company BAPEX Pharma, which is based in New Delhi. Elabscience, China supplied the ELISA kits (MBP, cAMP, CREB, TNF-, IL-1) (ELab Sciences, Wuhan, China). The rest of the substances employed in the experiment are also important to research. Before use, the drug and chemical solutions were freshly prepared. 6-OHDA was dissolved in 0.5 M HCL and DMSO and delivered at a dose of 8 g/2 µL/unilateral for 1–7 days. FSK was dissolved in water (containing 2% ethanol) and administered orally [21,26].

### 4.3. Experimental Protocol Schedule

**Figure 14:**
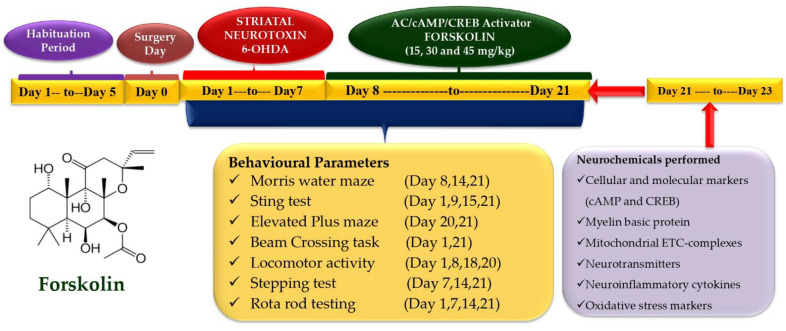
Experimental protocol schedule (behavioural and biochemical estimations).

The investigator undertook an unblended and well-known investigation. To eliminate the effect of circadian rhythm on the results, the animal experiment protocol was carried out between 9:00 a.m. and 1:00 p.m. The protocol trial lasted a total of 21 days. Thirty-six male Wistar rats (22 weeks old; 220–250 g) were divided into groups as follows: Group 1: naive (*n* = 6); Group 2: naive + FSK (45 mg/kg, p.o.) (*n* = 6); Group 3: 6-OHDA control (8 μg/2 μL/unilateral) (*n* = 6); Group 4: FSK (15 mg/kg, p.o.) + 6-OHDA (8 μg/2 μL/unilateral) (*n* = 6); group 5: FSK (30 mg/kg, p.o.) + 6-OHDA (8 μg/2 μL/unilateral) (*n* = 6); Group 6: FSK (45 mg/kg, p.o.) + 6-OHDA (8 μg/2 μL/unilateral) (*n* = 6). From day 1 to day 7, the 6-OHDA (8 μg/2 μL/unilateral) was infused stereotaxically through the intrastriatal route in groups 3, 4, 5 and 6. Subsequently, the FSK was given orally from day 8 and continued until day 21 in groups 4, 5 and 6 at a dose of 15, 30 and 45 mg/kg, respectively. On the last days, behavioural tests such as the (MWM), locomotor, grip strength, EPM, BCT, stepping test and rotarod were performed. The animals were euthanised after day 21 of the treatment, and their brains were separated to perform biochemical, inflammatory and neurochemical assessments (Figure 14).

### 4.4. Animal Model of 6-OHDA-Induced PD in Rats

All animals in experimental groups 3, 4, 5 and 6 were anaesthetised with Ketamine (70 mg/kg b.w) (i.p.) and Xylazine (10 mg/kg b.w) (s.c.). After shaving the scalp hair, we cut about 1–2 cm along from the anterior to the posterior to expose the scalp to measure and mark the striatum coordinates. Each animal was mounted on a stereotaxic apparatus, the skin overlying the skull was cut to expose it, and the coordinates for the striatum were measured accurately from bregma (anterior-posterior 0.5 mm, lateral 2.5 mm, 5 mm dorso-ventral from dura) with the tooth bar set at 0 mm (stereotaxic co-ordinates followed by Paxinos and Watson, 2013, 7th edition). After that, all animals in the experimental groups were lesioned by injecting (8 μg/2 μL 6-OHDA in 0.1% ascorbic acid-saline) into the right striatum. The cannula was inserted into the hole and fixed using dental cement. Then, the incision was stitched using absorbable suturing thread and a sterile needle. Cannula-implanted rats were housed individually in polyacrylic cages with warm clothes to avoid hypothermia-like conditions in surgical rats, and the room temperature was kept at 25 ± 3 °C. The rats were given special attention until they regained spontaneous movement following surgery. After surgery, milk and glucose water were kept in the cages to minimise physical trauma. To avoid sepsis, rats were given gentamycin (35 mg/kg) intraperitoneally (i.p.) for 3 days. On the sutures, lignocaine gel was applied to relieve pain. The sutures were dusted with Neosporin powder to avoid bacterial infection. The injections were made stereotaxically with the help of a Hamilton syringe through the burr holes made on the skull surface in the experimental groups. The injection rate was 1.0 μL/min, and the Hamilton syringe was kept in place for an additional 1 min before being slowly retracted [13].

### 4.5. Behavioural Parameters

#### 4.5.1. Morris Water Maze (MWM) for Memory and Cognition

The animals’ capacity to learn and retain spatial information was evaluated on days 5, 10 and 15 using the MWM. The animals were trained for 4 days (days 1, 2, 3 and 4) before the final trial, i.e., on days 5, 10 and 15 of the protocol schedule. Each trial had a ceiling time of 120 s and an interval between two trials of roughly around 120 s. TSTQ (time spent in the target quadrant zone) was also measured to find out how long it takes to find the secret platform. The amount of time spent in the “targeted” quadrant was linked to how well newly learned information was preserved in the long-term memory [51].

#### 4.5.2. String Test for Grip Strength

The animals’ grip strength was measured using a grip strength meter (Hague Ave, Columbus, OH 43204 USA). A grip strength test was performed on day 1, day 9, day 15 and day 21 as per the protocol. When the paw was released from the platform, the highest force applied was measured in kilogram-force (Kgf) [52].

#### 4.5.3. Elevated Plus Maze Test (EPM) Task for Working Memory

The rats’ learning and memory were assessed using the EPM, a behavioural model in which the stimulus is external to the body. EPM was carried out across 2 days, on the 13th and 14th days of the protocol schedule. On the 13th day, the animals were positioned on the open ends of their arms, with their backs on the centre platform. TL was measured on the 13th day, related to the acquisition trial, and again 24 h later on the 14th day of the protocol schedule, which corresponded to the retention trial. The time it took the animal to move into any of the covered arms with all four legs was defined as TL [53].

#### 4.5.4. Beam Crossing Task (BCT) for Neuromuscular Coordination

Gait irregularities and the number of times the foot slipped while walking was measured using the NBW test device. Before the experiments, all animals were trained for 7 days on the NBW. The length of time it took the animal to get from one location to another and the number of falls it experienced were recorded for each trial [54].

#### 4.5.5. Actophotometer for Locomotion

The animals’ ability to move about of their own accord was measured in the open field weekly. The open field apparatus consists of 25 black and white boxes that measured how many squares were crossed during movement and how much this increased from the 1st to the 21st day. The duration of the experiment was 5 min [55].

#### 4.5.6. Stepping Test for Akinesia

Forelimb akinesia was assessed by a stepping test in rats 3 weeks post-lesion. Each forelimb’s time to initiate stepping, the length of each stride and the adjustment time were measured throughout the stepping test. Experiments were performed on days 7, 14 t and 21 as specified in the protocol. The experimenter measured the time, not the fixed movement of the forelimb. The amount of time that passed before the rat started moving the forelimb that the experimenter had not fixed was measured. An initiation time of this length was established, with 180 s serving as the cut-off. When the rats were moved forward and backward along the table at a rate of 90 cm every 5 s, their free forelimbs had to step with the movement of the investigator to maintain balance. The adjusting steps were recorded as the total number of free forepaw steps while maintaining balance. The two arms were put to the test alternately [56].

#### 4.5.7. Rotarod Test for Grip and Neuromuscular Strength

Animals were tested on their motor skills and coordination using the rotarod device. Before undergoing the actual experimentation, each animal was trained for 7 days. The experiment was conducted on January 1st, 7th, 14th and 21st when animals were placed on the spinning rod at a speed of 25 revolutions per minute. The time was limited to a maximum of 180 s [57].

### 4.6. Quantification of Biochemical Parameters

On the 22nd day of the treatment, the rats were sacrificed by decapitation, and their brains were removed, washed and stored in an ice-cold isotonic saline solution. At room temperature, the brain was homogenised 10 times (*w*/*v*) with 0.1 M potassium phosphate buffer (7.4). The supernatant was collected after centrifuging the homogenate for 15 min at 10,000× *g*, and aliquots were used for biochemical examination [58].

#### 4.6.1. Estimation of Cellular and Molecular Markers

##### Analysis of Myelin Basic Protein (MBP) Level

A commercial ELISA kit (E-EL-R0010/MBP/E lab sciences; Wuhan, Hubei, China) was used to detect the amount of MBP in the sample. The calculated values were displayed as a function of protein content in µg/mg [59].

#### 4.6.2. Estimation of Mitochondrial ETC-Complexes’ Enzymes Activity

##### Preparation of Crude Mitochondrial Fraction from Rat Whole Brain Homogenate

A crude form of the mitochondrial fraction was prepared. The pellet formed during the PMS preparation procedure was mixed in a 1:10 proportion with a 0.1 M sodium phosphate buffer (pH 7.4) after 60 min of stirring at 4 °C. For 30 min, the mixture was centrifuged at 16,000× *g* at 0 °C. The pellets were resuspended in a 250 mmol/L sucrose solution added to the original buffer. The crude mitochondrial fraction in the buffered sucrose solution was prepared three times using the centrifugation and resuspension method [60].

##### Analysis of Complex-1 Enzyme Levels (NADPH Dehydrogenase)

The post-nuclear supernatant was collected from mechanically homogenised sections of adult rat brains in a buffer containing 10 mMTris-HCl pH 7.2, 225 mannitol, 75 saccharose and 0.1 mM EDTA after centrifugation (600 g, 4 °C, 20 min). The rate of NADH oxidation at 340 nm was utilised to estimate Complex I activity at 37 °C for 3 min by spectrophotometry [61].

##### Analysis of Complex-II Enzyme Level (Succinate Dehydrogenase/SDH)

The gradient fraction of 50 μL of the homogenate was mixed with 0.3 mL of sodium succinate solution. Following a 10–20 min incubation period at 37 °C, 0.1 mL of p-iodonitrotetrazolium violet (INT) solution was added to the mixture, which was then incubated for another 10 min. After adding 1 mL of a 5:5:1 (*v*/*v*/*w*) solution of ethyl acetate, ethanol and trichloroacetic acid (TCA) to the reaction mixture and centrifuging at 15,000× *g* rpm for 1 min, the absorbance at 490 nm was measured (Shimadzu, UV-1700). The amount of lowered INT mol/mg protein was reported [28].

##### Analysis of Complex-V Enzyme Level (ATPase)

Small homogenate aliquots were sonicated in ice-cold per-chloric acid to deactivate the ATPases (0.1 N). Before analysis, ATP-containing supernatants were centrifuged (14,000× *g*, 4 °C, 5 min), neutralised with 1N NaOH and frozen at −80 °C. We measured the amount of ATP in the supernatants using a reverse-phase high-performance liquid chromatography (HPLC) device (Perkin Elmer). A 100 mM KH2PO4 buffer solution (pH 6.0), a flow rate of 1.2 mL/min, a column temperature of 25 °C and a detection wavelength of 254 nm were used in separation. The ATP reference solution was created using the same approach as the dissolving standard (Sigma, St. Louis., MO, USA) [62].

##### Estimation of cAMP and CREB Levels

The immunoreactivity of cAMP and CREB was measured in homogenised tissue using ELISA’s commercial kits (E-EL-R0056/cAMP; E-EL-R0289/CREB, Elabsciences, Wuhan, Hubei, China). The values were expressed as pg/mL [25].

#### 4.6.3. Estimation of Neuroinflammatory Cytokines

##### Analysis of TNF-α, IL-1β, IL-6 and IL-10 Levels

Using a diagnostic kit (E-EL-R0019/TNF-α; E-EL-R0012/IL-1β; E-EL-R0014/IL-6; E-EL-R0016/IL-10, E lab sciences, Wuhan Hubei, China), the levels of TNF-α, IL-1β, IL-6 and IL-10 were determined. The kit instructions were strictly followed, and the reagents and samples were prepared accordingly. The optical density of the reaction mixture was measured in a microtiter plate at 450 nm [63,64,65].

#### 4.6.4. Neurotransmitter Measurement

##### Neuronal GABA and Glutamate Levels

The amounts of GABA and glutamate were determined using HPLC and an ECD. A standard Waters system with a high-pressure isocratic pump, a 20 μL manual sample injector valve, a C18 reversed-phase column and a UV detector was integrated with an ECD. The mobile phase comprised 22% methanol, 25% EDTA and 100% anhydrous disodium hydrogen phosphate (pH 6.5). The experimental electrochemical conditions were +0.65 V and the sensitivity ranged from 5 to 50 nA. The separation flow rate was 1.2 mL/min, and the column temperature was maintained at 40 °C. To manually inject samples (20 μL), a rheodyne valve injector was employed. On the day of the experiment, frozen brain samples were thawed and homogenised in 0.2 M perchloric acid. After that, the samples were exposed to an anteroposterior 12,000 g for 15 min. The supernatant was injected into the HPLC sample injector after being derivatised with OPA/-ME (o-pthalaldehyde/-mercaptoethanol) and filtered using 0.22 m nylon filters. Data were recorded and analysed using Waters HPLC’s Breeze (version 3.2). The concentrations of amino acids were calculated by extrapolating a standard curve with a concentration standard between 10 and 100 ng/mL. A naive group was used for comparison, and the results were provided as a percentage [66].

##### Neuronal DA Level

DA levels in the brain were measured using high-performance liquid chromatography (HPLC) with an electrochemical detector (ECD). A Waters standard system was used during the research. This system includes a high-pressure isocratic pump, a 20 μL manual sample injector valve, a C18 reversed-phase column and an ECD. The mobile phase was an acetonitrile-sodium citrate buffer (pH 4.5) mixture (87:13, *v*/*v*). The sodium citrate buffer was composed of 10 mM citric acid, 25 mM NaH2HPO4, 25 mM EDTA (ethylene diamine tetra-acetic acid) and 2 mM 1-heptane sulfonic acid. The electrochemical parameters of the experiment were +0.75 V, with a 5–50 nA sensitivity. A flow rate of 0.8 mL/min was applied for this separation. Each sample was injected by hand at a volume of 20 μL. The experiment’s homogenising solution contained 0.2 M perchloric acid, which was used to defrost the frozen brain samples before homogenisation. After that, the samples were centrifuged at 12,000× *g* for 5 min. The supernatant was filtered through 0.22-mm nylon filters before being injected into the HPLC sample injector. Breeze software was used to collect and analyse the data [67].

##### Neuronal (Ach) Level

A diagnostic kit measured Ach levels (Krishgen diagnostics, Mumbai, India). All reagents and samples were made according to the instructions included in the kit. A microtiter plate was used to take readings of the optical density of the reaction mixture at 540 nm [59].

#### 4.6.5. Assessment of Oxidative Stress Markers

##### Analysis of Lactate Dehydrogenase (LDH) Level

A diagnostic kit (Coral Diagnostics, India) was used to measure the lactate dehydrogenase activity in a rat brain homogenate; the results were reported in international units per litre (IU/L) [68].

##### Analysis of Superoxide Dismutase (SOD) Level

The spectrophotometric detection of the auto-oxidation of epinephrine at a pH of 10.4 was used to calculate SOD activity. This technique found 0.02 mL of epinephrine in the brain homogenate supernatant (0.2 mL). The absorbance was determined using spectrophotometry at 480 nm after 5 min. SOD activity was reported as % of the control value [55].

##### Analysis of Catalase Level

An assay for CAT activity was performed by adding 0.1 mL of supernatant to 1.9 mL of 50 mM phosphate buffer in a cuvette (pH 7.0). We began the process by adding 1.0 mL of freshly produced 30 mM H_2_O_2_. Spectrophotometric analysis at 240 nm was used to determine the rate of H_2_O_2_ breakdown. CAT activity was measured in µM/H_2_O_2_ decomposition/minute [69].

##### Analysis of Acetylcholinesterase (AChE) Level

We quantitatively evaluated the acetylcholinesterase activity in the brain following the protocol. A total of 0.05 mL of supernatant, 3 mL of 0.01 M sodium phosphate buffer (pH 8), 0.10 mL of acetylthiocholine iodide and 0.10 mL of dithiothreitol n-butyrate (DTNB) were combined to create the test combination (Ellman reagent). The absorbance shift was immediately measured spectrophotometrically at 412 nm. Enzymatic activity in the supernatant was measured in M/mg protein units [70].

##### Analysis of Myeloperoxidase (MPO) Level

The samples of midbrain tissue were rapidly removed and frozen in liquid nitrogen. We measured out an appropriate amount of frozen tissue, washed it twice in phosphate buffer, pH 6.0 at 4–8 °C, and then homogenised it (IKA Homogenizer, Staufen, Germany) in a solution of 0.5% hexadecyltrimethylammonium bromide (HTAB) mixed in 50 mM potassium phosphate buffer (pH 6). The sample underwent centrifugation for 20 min at a temperature of 4 °C and 10,000 revolutions per minute. The samples were sonicated for 20 s, then frozen and thawed thrice. A 2.9 mL solution of 50 mM potassium phosphate buffer at pH 6 containing 0.167 mg/mL of O-dianisidine hydrochloride and 0.0005% H_2_O_2_ was allowed to react with a 0.1 mL aliquot of the supernatant or standard (Sigma, Germany). After 5 min, the reaction was stopped with 0.1 cc of 1.2 M hydrochloric acid. The absorbance rate at 460 nm was measured using a spectrophotometer (Cecil 9000, Cambridge, UK). MPO activity was expressed as milli-units (mU) per gram weight of moist tissue [71].

##### Analysis of Reduced Glutathione (GSH) Level

After 1 mL of supernatant was precipitated with 1 mL of 4% sulfosalicylic acid, cold digestion took place at 4 °C for 1 h. The samples were centrifuged at 1200× *g* for 15 min. The supernatant was diluted to 1 mL with phosphate buffer (0.1 M, pH 8) and 5,5′-dithiobis-(2-nitrobenzoic acid) (DTNB). The newly produced yellow colour at 412 nm was instantly measured using a spectrophotometer. The GSH concentration in the supernatant was reported as mM per mg of protein [72].

##### Analysis of Nitrite Level

Colorimetric assays using the Greiss reagent (0.1% N-(1-naphthyl) ethylene diaminedihydrochloride, 1% sulfanilamide and 2.5% phosphoric acid) can measure nitric oxide concentrations. The absorbance at 540 nm is determined spectrophotometrically after mixing equal parts supernatant and Greiss reagent and incubating the mixture for 10 min at room temperature in the dark. The nitrite content of the supernatant was determined using a sodium nitrite standard curve and reported in M/mg protein units [73].

##### Analysis of Malondialdehyde (MDA) Level

Quantifying MDA in brain homogenates was performed by measuring the quantity of MDA at 532 nm following its reaction with thiobarbituric acid. Amounts of MDA were reported in nM/mg protein [26].

##### Analysis of Protein Carbonyl (PC) Level

An equal volume of 20% TCA was added to the tissue homogenate (0.25 mL). After adding 0.5 mL of 20% TCA, 0.25 mL of 10 mM 2,4-dinitrophenylhydrazine (DNPH) in 2.0 M HCl was added, and the mixture was left at room temperature for 1 h while being shaken every 10–15 min. We discarded the supernatant and washed the pellet three times with 1 mL of ethanol: ethyl acetate (1:1) to get rid of the free reagent. After letting the samples sit for 10 min, centrifugation was performed, and the resulting supernatant was thrown away. Within 15 min at 37–50 °C, the precipitated protein was redissolved in a guanidine hydrochloride solution and centrifuged at 11,000× *g* for 5 min to remove insoluble material. Spectrophotometric analysis of the carbonyl concentrations was performed at 370 nm (Shimadzu-1601, Nagoya, Japan). Using a molar extinction coefficient of 22 × 10^3^ M^−1^ cm^−1^, the data were represented as nM/mg protein [74].

##### Analysis of Total Glutathione Level

The total GSH level was measured in brain tissue homogenates with 20 volumes of ice-cold Tris-HCl buffer (20 mM, pH 7.4) containing 2 mM EDTA, followed by adding a similar volume of 10% trichloroacetic acid. After centrifugation (1000× *g*, 15 min), the supernatant was mixed with six volumes of ice-cold diethyl ether, followed by the evaporation of diethyl ether with N_2_ gas. This step was repeated five times to remove trichloroacetic acid. The final samples were diluted with five volumes of ice-cold phosphate buffer (10 mM, pH 7.4). In the next step, 730 µL of 10 mM phosphate buffer (pH 7.4) containing 0.27 mM NADPH and 0.8 U GSH reductase was added to 20 µL of the diluted samples. After incubation for 5 min at 37 °C, 50 µL of 0.1 M phosphate buffer (pH 7.4) containing 4 mg/mL 5,5′-dithiobis (2-nitro) benzoic acid was added. The formation of p-nitrophenol was determined by measuring its absorbance at 412 nm for 6 min after adding 5,5′-dithiobis (2-nitro) benzoic acid [75].

##### Analysis of Hydrogen Peroxide (H_2_O_2_) Level

50 mg of brain tissue fragments were homogenised with 2 mL potassium chloride (1.15%) solution. The tissue homogenate was diluted to a final volume of 10 μL before being combined with either 90 μL of physiologically buffered saline (pH 7.0) and 100 μL of horseradish peroxidase (1 U/mL) with 400 μL of homovanillic acids (HRP + HVA assay), or 90 μL of PBS and 100 μL of 1 U/mL horseradish peroxidase (HRP assay). After homogenisation, the samples were incubated at 37 °C for an hour. A total of 300 μL of phosphate-buffered saline (PBS) and 125 µL of 0.1 M glycine-NaOH buffer (pH 12.0) containing 25 mM EDTA (ethylenediaminetetraacetic acid) were added to each homogenate sample. An excitation wavelength of 312 nm and an emission wavelength of 420 nm were settled upon (Perkin Elmer Luminescence Spectrometer, Beaconsfield, UK) [76].

### 4.7. Histopathological Analysis of Striatum

Once the experimental schedule was completed, animals were anesthetised with sodium barbiturates at a 270 mg/kg dose, i.p., before decapitation. The midbrain was separated from the whole brain and cleaned with PBS. As directed, the sections were immersed in 4% paraformaldehyde in PBS at pH = 7.4. Tissue was embedded in paraffin wax and kept at 37 °C. A rotary microtome was used to section the paraffin wax blocks into 4–5 mm thicknesses. Morphological analysis was carried out with the help of staining with haematoxylin and eosin dye on the sections at 100× magnification. Neuronal cell density, the number of degenerating dopaminergic neurons and the formation of Lewy bodies were observed in the stained slide using a fluorescent microscope (MOTICAM-Ba310 image plus 2.0) [62].

### 4.8. Statistical Analysis

To compare the results of the various experimental groups, we used two-way ANOVA with post-hoc Bonferroni’s test and one-way ANOVA repeated measures with post-hoc Tukey’s multi-comparison test to evaluate the data generated. Multiple behavioural indicators were analysed using two-way ANOVA. However, one-way ANOVA was employed to examine the neurochemical parameters in brain homogenate, demyelination volume and TSTQ task. The statistical significance was set at 0.01. The sample size was determined to ensure that the data followed a normal distribution. The Windows version of GraphPad Prism 5.03 was used for the statistical analysis (GraphPad Software, San Diego, CA, USA). The mean and the standard deviation (SD) are the only statistics supplied.

## 5. Conclusions

In conclusion, we found that the intracerebroventricular injection of 6-OHDA causes unilateral lesions with PD-like behavioural, morphological, biochemical and neurochemical characteristics. After 8–21 days of treatment with FSK, the effects of 6-OHDA on behaviour, biochemistry and the nervous system are reversed, which leads to the control of CREB-mediated signalling and the direct activation of D1 in the striatum. The inclusion of the test medicine FSK in the cAMP/PKA/CREB pathway, as well as its antioxidant, anti-inflammatory and neuromodulatory actions, are all possibilities for the processes that could be responsible for the amelioration of PD-like neuropathological abnormalities. These results led researchers to speculate that FSK might be useful as a therapeutic component in the clinical management of PD. As a result, our study suggests and opens up the possibility for medicinal chemists to develop more effective and viable novel FSK analogues for treating neurological dysfunctions.

## Data Availability

All data generated or analysed during this study are included in this article. There are no separate or additional files.

## Sample Availability

Samples of the compounds are not available from the authors.

## Abbreviations

Aβ	Amyloid beta
AC	Adenylcyclase
ACh	Acetylcholine
AChE	Acetyl cholinesterase
ANOVA	Analysis of variance
ATP	Adenosine triphosphate
BCT	Beam crossing task
BDNF	Brain-derived neurotrophic factor
BG	Basal Ganglia
cAMP	Cyclic adenosine monophosphate
CAT	Catalase
CREB	cAMP-responsive element-binding protein
DA	Dopamine
DNA	Deoxy ribonucleic acid
DTNB	5,5′-dithiobis-(2-nitrobenzoic acid)
ECD	Electron capture detector
EDTA	Ethylenediaminetetraacetic acid
ELISA	Enzyme-linked immunosorbent assay
ELT	Escape latency test
EPM	Elevated plus maze
ERK1/2	Extracellular signal-regulated kinase 2
ETC	Electron transport chain
FSK	Forskolin
GABA	Gamma- amino butyric acid
GSH	Glutathione
HD	Huntington’s disease
H_2_O_2_	Hydrogen peroxide
HPLC	High-Performance Liquid Chromatography
IL-β	Interleukin beta
i-NOS	Inducible nitric oxide synthase
INT- p	Iodonitrotetrazolium
I.P.	Intraperitoneal
LA	Locomotor activity
LDH	Lactate dehydrogenase
LTP	Long-term memory potentiation
MBP	Myelin basic protein
MDA	Malondialdehyde
MPO	Myeloperoxidase
MPTP	Methyl-4-phenyl-1,2,3,6-tetrahydropyridine
MWM	Morris water maze
NADPH	Nicotinamide adenine dinucleotide phosphate
NBW	Narrow beam walk
NGF	Neuronal growth factor
6-OHDA	6-Hydroxydopamine
PAF	Plasminogen activating factor
PC	Protein carbonyl
PD	Parkinson’s disease
PKA	Protein kinase A
PI3K/Akt	phosphatidylinositol 3-kinase and protein kinase B
PMS	phenyl methyl sulphide
PSA-NCAM	polysialylated neuronal cell adhesion molecule
P.O	by mouth
ROS	Reactive oxygen species
S.C	Subcutaneous
SDH	Succinate dehydrogenase
SLA	Spontaneous locomotor activity
SNcp	Substanianigra pars compacta
SOD	Superoxide dismutase
TCA	Trichloroacetic acid
TH	Tyrosine hydroxylase
TL	Transfer latency
TNF-α	Tumour necrosis factor-α
TSTQ	Time spent in the target quadrant zone
